# Supplementary material for: Extensive gene content variation in the Brachypodium distachyon pan-genome correlates with population structure
Source: Nat Commun. 2017 Dec 19;8:2184. doi: 10.1038/s41467-017-02292-8 (PMC5736591; doi:10.1038/s41467-017-02292-8)
Supplement: Supplementary file 1 — Supplementary information [file 41467_2017_2292_MOESM1_ESM.pdf]

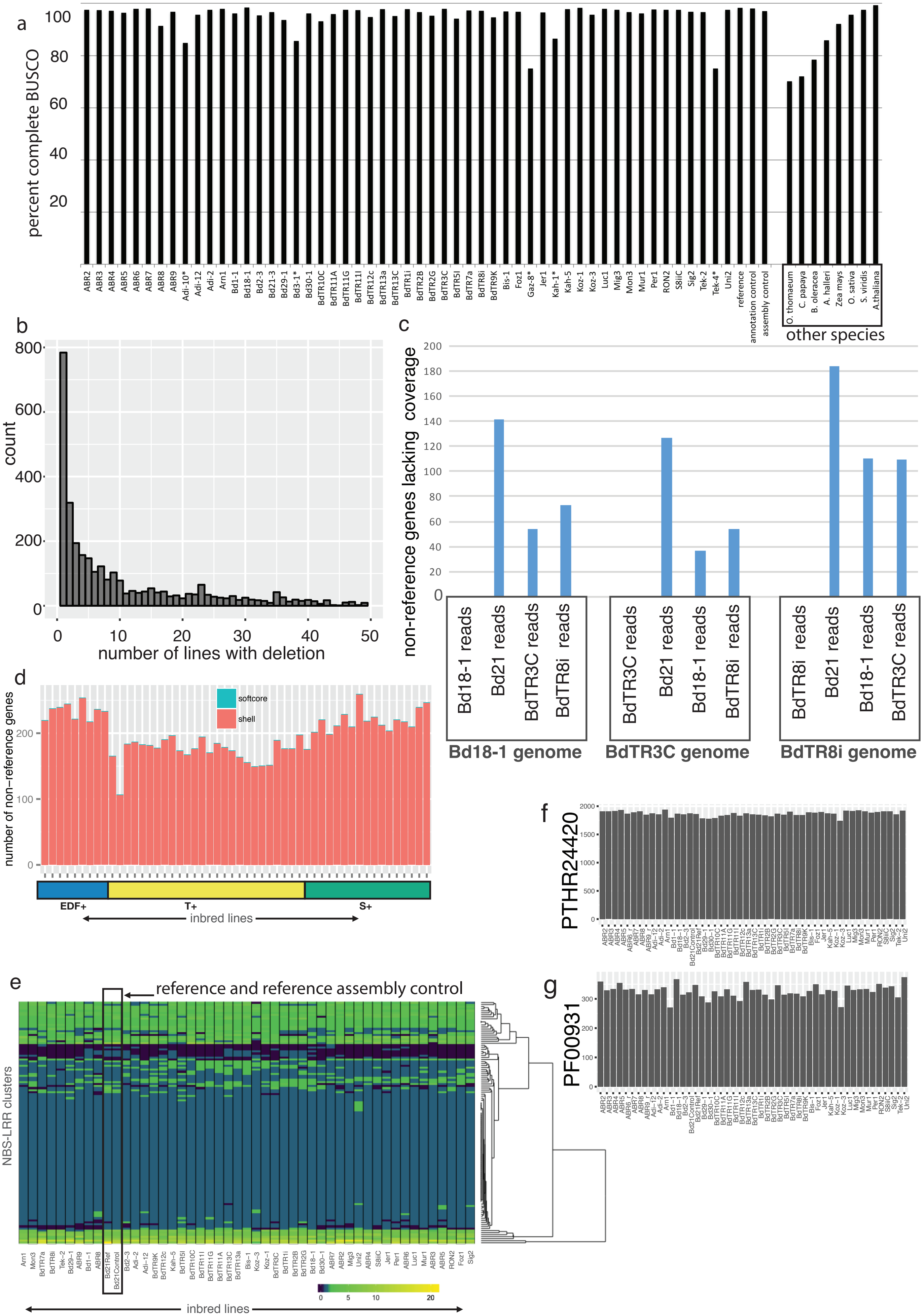

a
percent complete BUSCO
100
80
60
40
20
ABR2 ABR3 ABR4 ABR5 ABR6 ABR7 ABR8 ABR9 Adi-10* Adi-12 Adi-2 Arn1 Bd1-1 Bd18-1 Bd2-3 Bd21-3 Bd29-1 Bd3-1* Bd30-1 BdTR10C BdTR11A BdTR11G BdTR11I BdTR12c BdTR13a BdTR13C BdTR1i BdTR2B BdTR2G BdTR3C BdTR5I BdTR7a BdTR8i BdTR9K Bis-1 Foz1 Gaz-8* Jer1 Kah-1* Kah-5 Koz-1 Koz-3 Luc1 Mig3 Mon3 Mur1 Per1 RON2 S8iiC Sig2 Tek-2 Tek-4* Uni2 reference annotation control assembly control
O. thomaeum C. papaya B. oleracea A. halleri Zea mays O. sativa S. viridis A.thaliana
other species
b
count
800
600
400
200
0
0 10 20 30 40 50
number of lines with deletion
c
non-reference genes lacking coverage
200 180 160 140 120 100 80 60 40 20 0
Bd18-1 reads Bd21 reads BdTR3C reads BdTR8i reads
Bd18-1 genome
BdTR3C reads Bd21 reads Bd18-1 reads BdTR8i reads
BdTR3C genome
BdTR8i reads Bd21 reads Bd18-1 reads BdTR3C reads
BdTR8i genome
d
number of non-reference genes
200
100
0
softcore
shell
EDF+
T+
S+
inbred lines
e
reference and reference assembly control
NBS-LRR clusters
Arn1 Mon3 BdTR7a BdTR8i Tek-2 Bd29-1 ABR9 Bd1-1 ABR8 Bd21Ref Bd21Control Bd2-3 Adi-2 Adi-12 BdTR9K BdTR12c Kah-5 BdTR5I BdTR11I BdTR11G BdTR11A BdTR13C BdTR13a Bis-1 Koz-3 Koz-1 BdTR3C BdTR1i BdTR2B BdTR2G Bd18-1 Bd30-1 ABR7 ABR2 Mig3 Uni2 ABR4 S8iiC Jer1 Per1 ABR6 Luc1 Mur1 ABR3 ABR5 RON2 Foz1 Sig2
0 5 10 20
inbred lines
f
PTHR24420
2000
1500
1000
500
0
g
PF00931
300
200
100
0

Supplementary Figure 1. Estimating assembly completeness and accuracy. **a**, BUSCO percent completeness of all assemblies. Note that, with a few exceptions, the assemblies have the same number of BUSCO genes as the reference genome. All of the assemblies in this study have higher BUSCO completeness than some current reference genomes. **b**, Frequency of the absence of reference genes (Bd21 v2.1 annotation) among sequenced lines, estimated by lack of coverage by aligning short-reads from sequenced lines to the Bd21 reference assembly v2.0. **c**, Illumina short-read sequences from inbred lines Bd21 (the line used to produce the reference genome), Bd18-1, BdTR3C, and BdTR8i aligned to the Bd18-1, BdTR3C, and BdTR8i genome assemblies. Non-reference genes within the Bd18-1, BdTR3C, and BdTR8i assemblies were supported by their own raw reads while lacking coverage from alignment of the other 3 short-reads datasets, in particular the Bd21 reference line. **b-c**, Genes were considered lacking short read coverage or absent if <80% of the overall gene model was supported by alignments of more than three overlapping reads. **d**, Number of non-reference genes per inbred line. The EDF+ group, which is the most divergent from the group in which the reference resides (T+) contains the most shell genes per line. Lines from the T+ group harbor the lowest number of non-reference genes. Displayed non-reference genes are high-confidence as their transcripts do not map to the reference genome. **e**, 119 pan-genome clusters associated with reference genes previously identified as NBS-LRRs are plotted in rows with the number of sequences within each individual colored according to the scale below the plot. **f**, Number of genes annotated as PTHR24420 (Leucine rich repeat receptor-like protein kinase) and **g**, number of PF00931 NB-ARC protein, per inbred line.

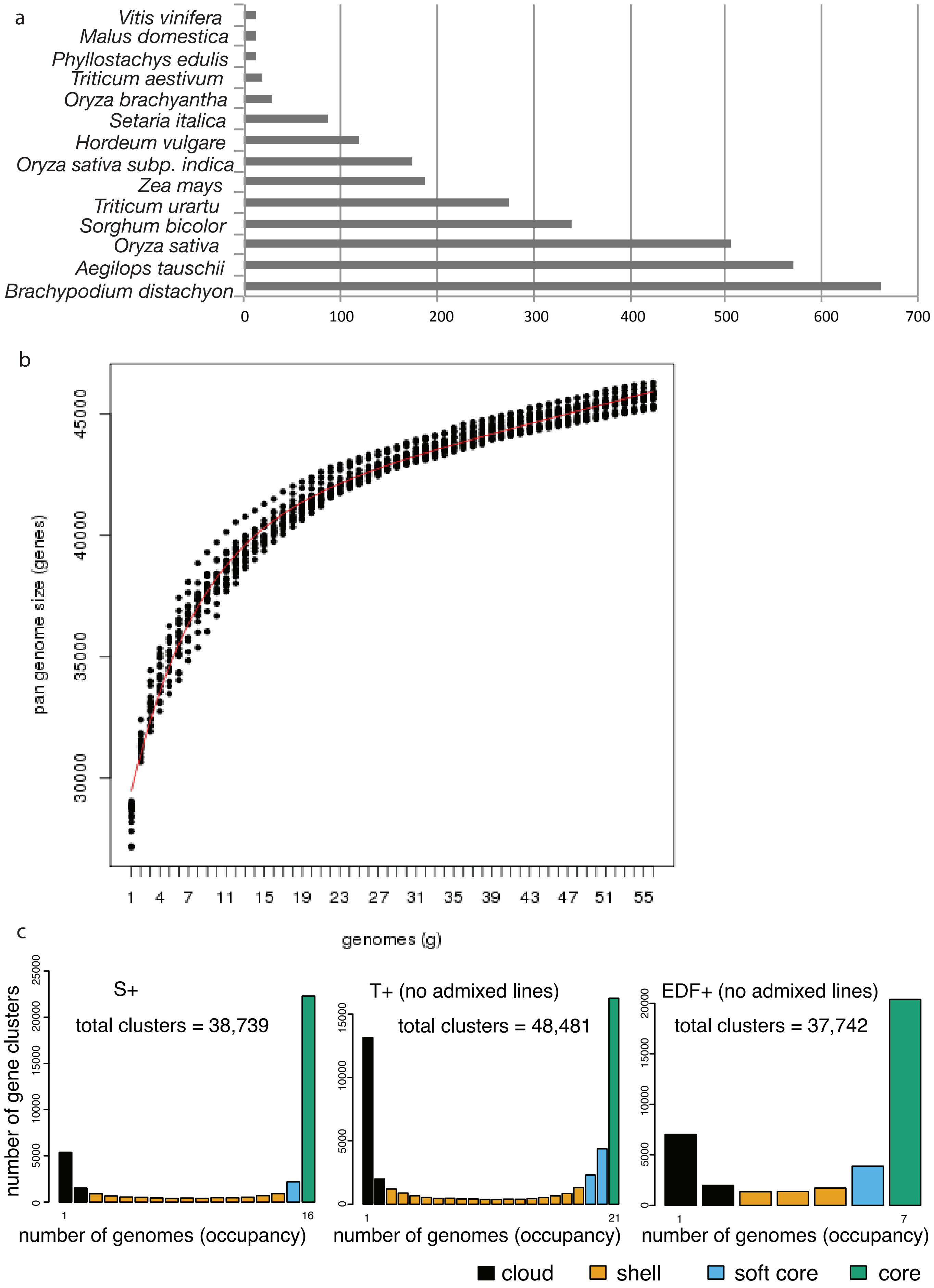
a
Vitis vinifera
Malus domestica
Phyllostachys edulis
Triticum aestivum
Oryza brachyantha
Setaria italica
Hordeum vulgare
Oryza sativa subp. indica
Zea mays
Triticum urartu
Sorghum bicolor
Oryza sativa
Aegilops tauschii
Brachypodium distachyon
0 100 200 300 400 500 600 700
b
pan genome size (genes)
30000 35000 40000 45000
1 4 7 11 15 19 23 27 31 35 39 43 47 51 55
genomes (g)
c
number of gene clusters
S+
total clusters = 38,739
T+ (no admixed lines)
total clusters = 48,481
EDF+ (no admixed lines)
total clusters = 37,742
number of genomes (occupancy)
cloud
shell
soft core
core

Supplementary Figure 2. Pan-genome sampling simulations and BLAST analysis of non-reference genes. **a**, Forty-two percent of high-confidence non-reference genes had BLAST matches to plant species and 78% of those hits were to species other than *B. distachyon* with the largest number of hits arising from the closely related grass, *Aegilops tauschii*, one of the three progenitors of bread wheat. **b**, We examined the relationship between the number of lines sampled and the size of the high-confidence (only includes pan-genes found in three or more lines) pan-genome. The simulations indicate that growth of the pan-genome slows as more lines are added, but does not plateau. This indicates that the full diversity of the species has not yet been captured. Each simulation adds the genomes in different order leading to different sequential increases in pan-genome size. **c**, Population group specific pan-genomes.

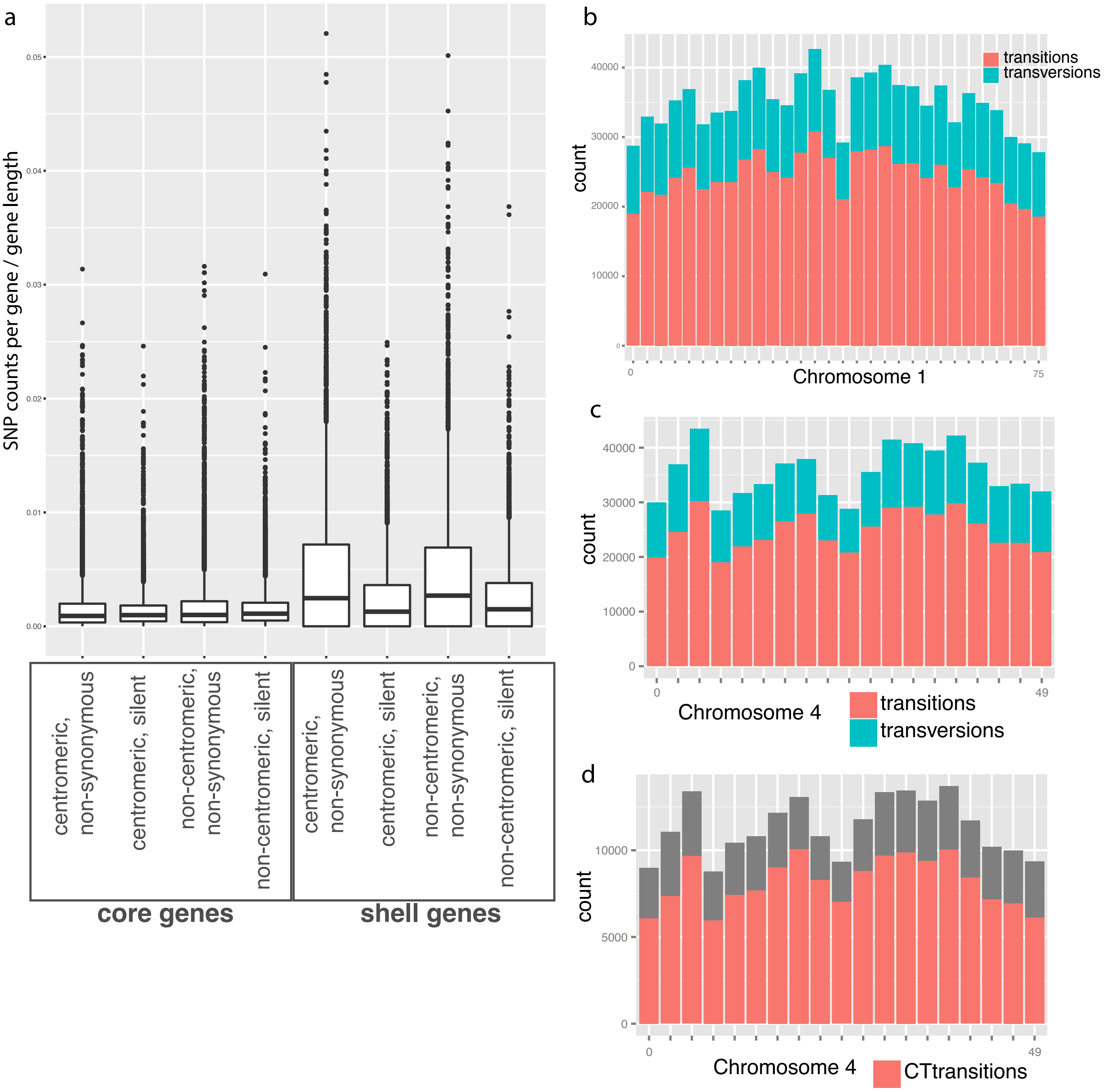

Supplementary Figure 3. Shell pan-genes have reduced sequence constraints. a, Shell genes have a higher frequency of non-synonymous and synonymous substitutions than core genes (number of respective differences relative to the reference, normalized according to gene length). However, there is no obvious difference between SNP frequency within genes between peri-centromeric regions as compared to distal regions of the chromosome. b, Higher frequency of SNPs are observed in peri-centromeric regions versus distal chromosome regions, particularly transitions, for representative chromosome 1 (Bd1). c, Similar pattern as panel (b) but for chromosome 4 (Bd4). d, Amongst transitions, C -> T transitions are elevated in peri-centromeric regions compared to distal regions.

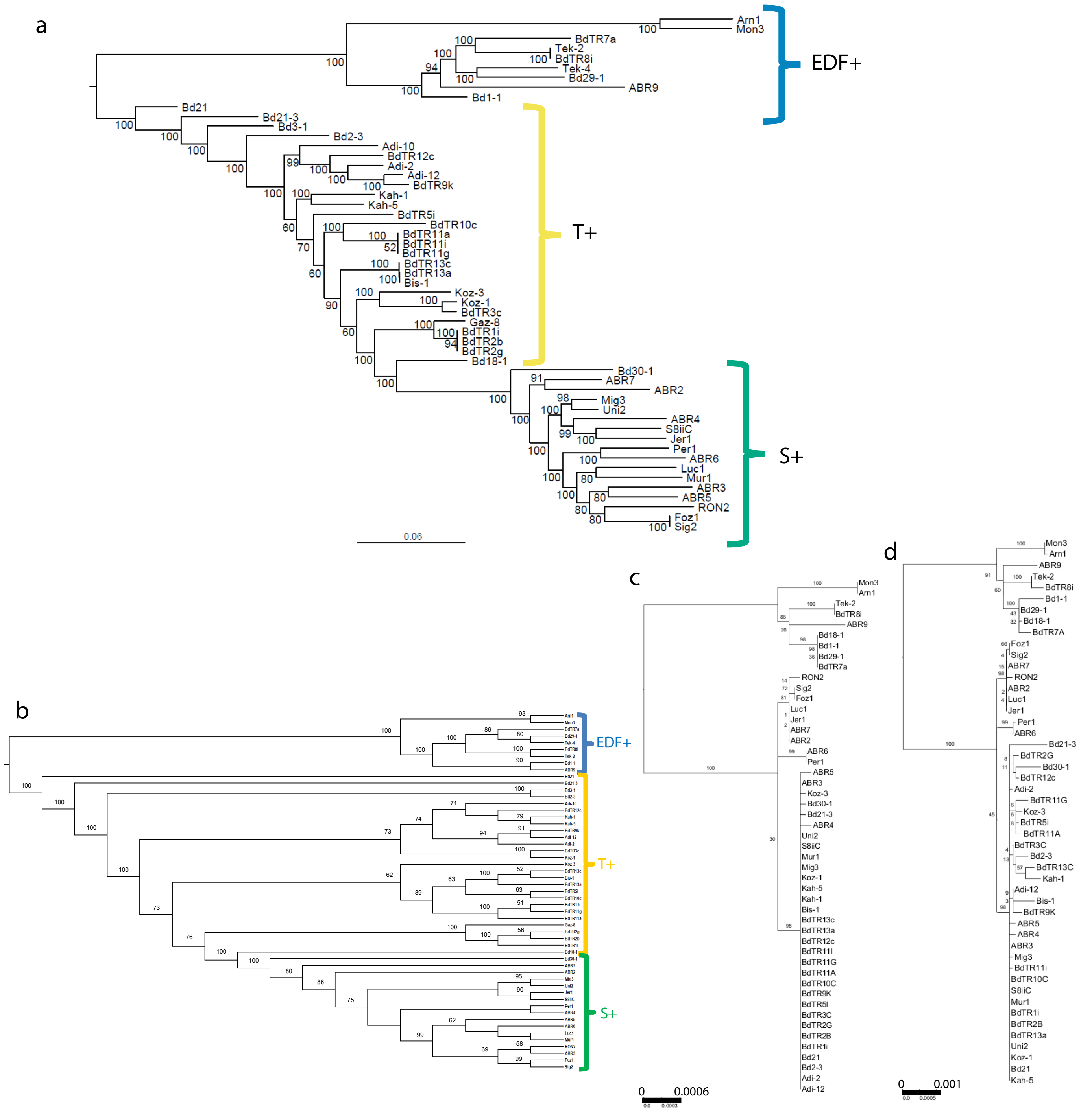

Supplementary Figure 4. Genome-wide SNP tree and high resolution gene trees from genome assemblies.
a, Maximum likelihood RAxML phylogenetic tree based on >3 million SNPs showing the relationships among 53 *B. distachyon* lines. Bootstrap support values are indicated on branches. The order of lines is the same as shown in figure 4a but the branch lengths are proportional to relatedness. Scale bar represents substitutions per site.
b, SVDquartets + Paup* tree based on >3 million SNPs showing the relationships among 53 *B. distachyon* lines. Note that the topology of the tree is very similar to that of the ML (RAxML) tree. Both trees show full agreement and support for the main clades and subclades, differing only in the reconstruction of few recently evolved and less supported lineages. Bootstrap support values >50% are indicated on the tree cladogram branches. **c,d**, Phylogenetic analysis of the BdVRN1 gene using variants identified from the genome assemblies is superior to the analysis using variants detected by read mapping to the reference genome. PHYML phylogenetic trees of BdVRN1 constructed from 122 variants identified by read mapping (**c**) and from 194 variants identified from the assembled genomes (**d**). Only three of the read mapping variants were not contained in the set of variants detected from the assembled genomes. Note that the tree based on the genome assemblies (**d**) has higher bootstrap support and higher resolution of closely related alleles than the gene tree based on read mapping (**c**). Branch lengths are proportional to substitutions per site (see scale bar).

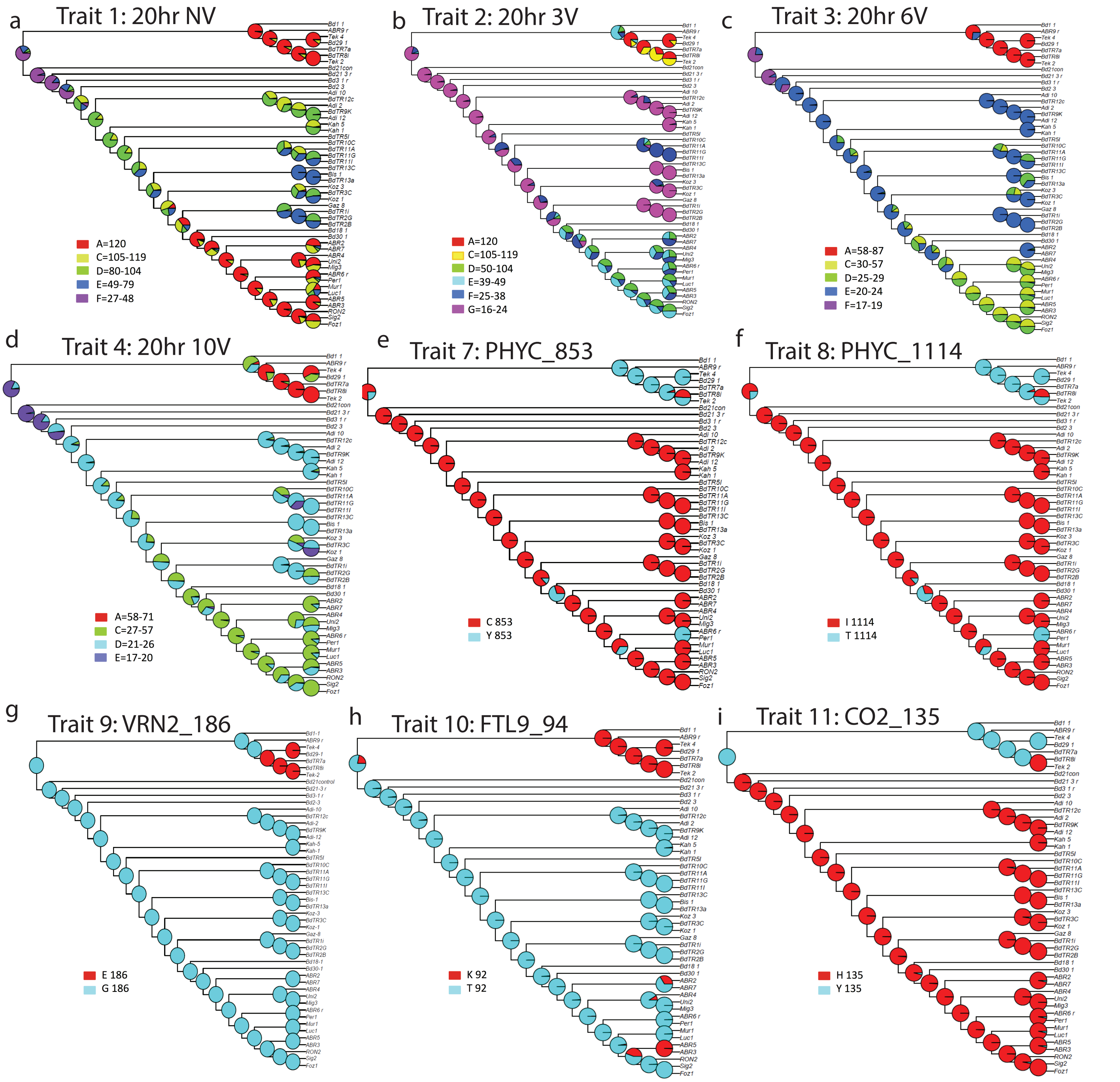

Supplemental Figure 5. Mapping the evolution of flowering traits on the *B. distachyon* SNP tree. Introgressed accessions Arn1 and Mon3 were pruned from the *B. distachyon* SNP tree with using the drop.tip option of the R package. A Maximum Likelihood (ML) mapping was performed with the program Bayes Traits. The ML searches were based on 1000 iterations using multistate and binary characters. The program inferred the probabilities of ancestral states at the tree nodes for each trait. Color codes of trait states are indicated in the corresponding charts. Trait numbers and codes correspond to those explained in Supplementary Table 3. ML mapping of flowering traits 20h NV (**a**), 20h 3V (**b**), 20h 6V (**c**), 20h 10V (**d**), and molecular variants in the PHYC-853 (**e**), PHYC_1114 (**f**), VNR2_186 (**g**), FTL9_94 (**h**), CO2_135 (**i**), CO2_152 (**j**), CO2_217 (**k**), CO2_235 (**l**), CO2_250 (**m**), FTL1_92 (**n**), FTL13_7 (**o**), FUL2_19 (**p**), FUL2_184del (**q**), PPD1_283 (**r**), PPD1_737 (**s**), VRN1_intron1_4kb (**t**), VRN1_intron1_6kb (**u**) genes.

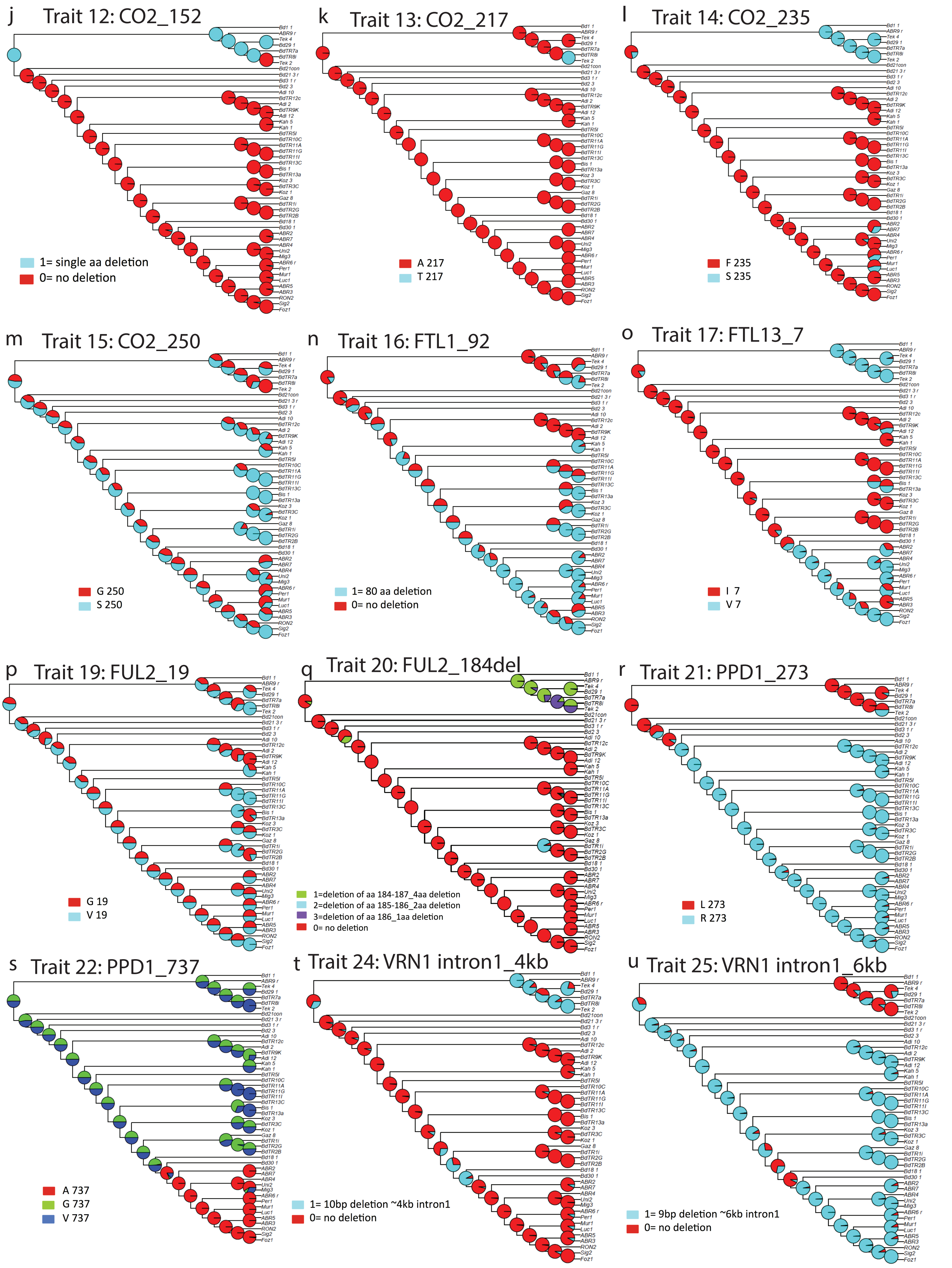

Supplemental Figure 5, continued.

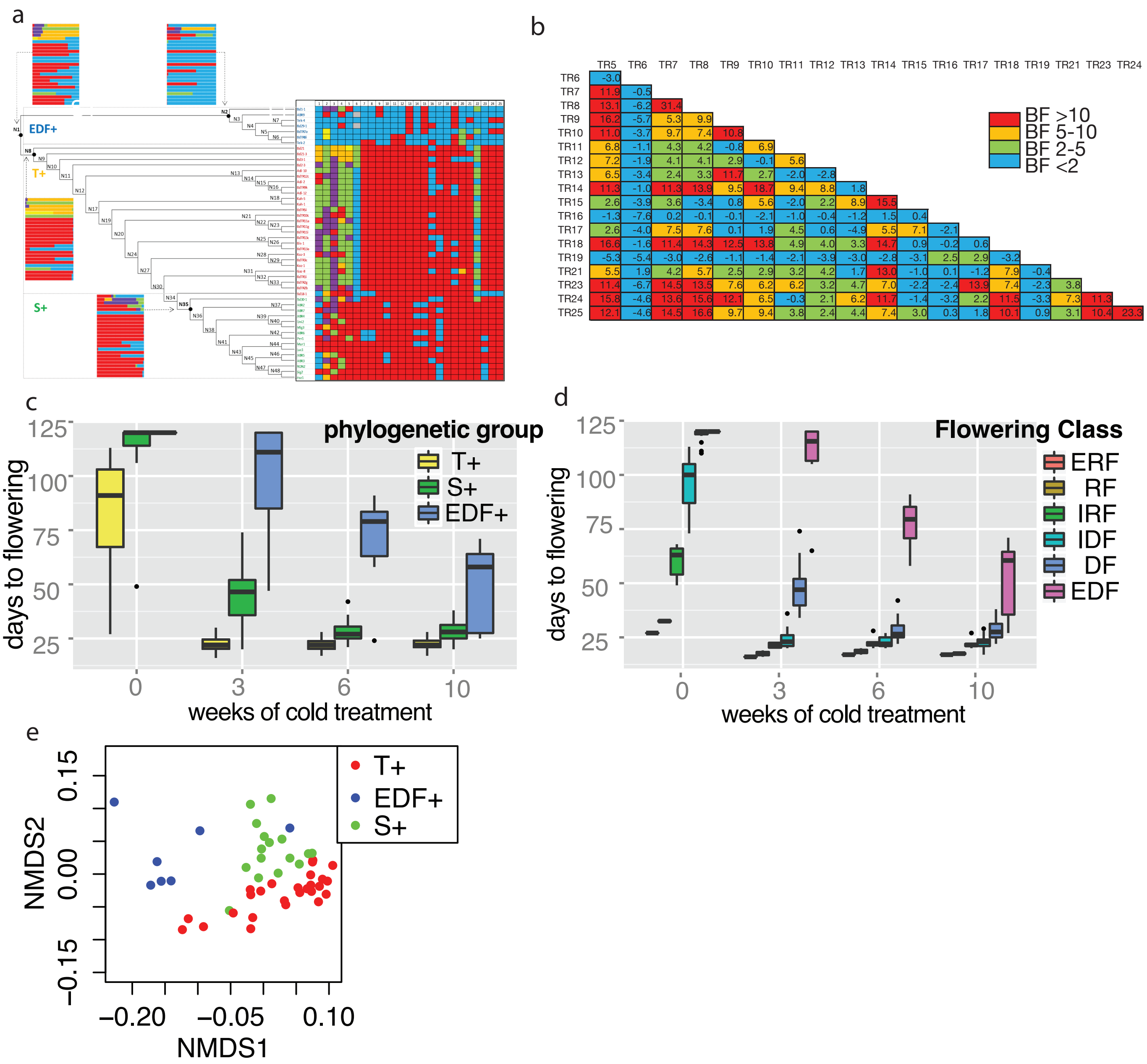

	TR5	TR6	TR7	TR8	TR9	TR10	TR11	TR12	TR13	TR14	TR15	TR16	TR17	TR18	TR19	TR21	TR23	TR24
TR6	-3.0																	
TR7	11.9	-0.5																
TR8	13.1	-6.2	31.4															
TR9	16.2	-5.7	5.3	9.9														
TR10	11.0	-3.7	9.7	7.4	10.8													
TR11	6.8	-1.1	4.3	4.2	-0.8	6.9												
TR12	7.2	-1.9	4.1	4.1	2.9	-0.1	5.6											
TR13	6.5	-3.4	2.4	3.3	11.7	2.7	-2.0	-2.8										
TR14	11.3	-1.0	11.3	13.9	9.5	18.7	9.4	8.8	1.8									
TR15	2.6	-3.9	3.6	-3.4	0.8	5.6	-2.0	2.2	8.9	15.5								
TR16	-1.3	-7.6	0.2	-0.1	-0.1	-2.1	-1.0	-0.4	-1.2	1.5	0.4							
TR17	2.6	-4.0	7.5	7.6	0.1	1.9	4.5	0.6	-4.9	5.5	7.1	-2.1						
TR18	16.6	-1.6	11.4	14.3	12.5	13.8	4.9	4.0	3.3	14.7	0.9	-0.2	0.6					
TR19	-5.3	-5.4	-3.0	-2.6	-1.1	-1.4	-2.1	-3.9	-3.0	-2.8	-3.1	2.5	2.9	-3.2				
TR21	5.5	1.9	4.2	5.7	2.5	2.9	3.2	4.2	1.7	13.0	-1.0	0.1	-1.2	7.9	-0.4			
TR23	11.4	-6.7	14.5	13.5	7.6	6.2	6.2	3.2	4.7	7.0	-2.2	-2.4	13.9	7.4	-2.3	3.8		
TR24	15.8	-4.6	13.6	15.6	12.1	6.5	-0.3	2.1	6.2	11.7	-1.4	-3.2	2.2	11.5	-3.3	7.3	11.3	
TR25	12.1	-4.6	14.5	16.6	9.7	9.4	3.8	2.4	4.4	7.4	3.0	0.3	1.8	10.1	0.9	3.1	10.4	23.3

Supplementary Figure 6. Correlation between flowering time traits and genetic variation. **a**, Mapping the evolution of flowering traits on the *B. distachyon* SNP tree. Introgressed accessions Arn1 and Mon3 were pruned from the tree using the drop.tip option of the R package APE. A Maximum Likelihood (ML) mapping was performed with the program Bayes Traits. The ML searches were based on 1000 iterations using multistate and binary characters. The program inferred the probabilities of ancestral states at the tree nodes for each trait. Color-coded matrix based on mapping all trait values to discrete state categories. Color codes of trait states are indicated in the corresponding charts. Trait numbers and codes correspond to those explained in Supplementary Table S3. **b**, Correlation of flowering time, latitude and polymorphisms in flowering genes. Bayes Factor (BF) tests for potential correlated evolution of the 19 binary traits (flowering time class, latitude and 17 flowering time genes) shown in (Figure 4c-e) indicated a very strong (BF values >10) and strong (BF = 5-10) evidence of correlation for 13 traits (7-14, 18, 21, 23-25) with flowering class (trait 5) and to each other, and positive evidence (BF = 2-5) for two other traits (15, 17). Conversely, all 18 examined traits showed weak evidence (BF <2) of correlated evolution with latitude (trait 6). Explanation of traits is found in supplementary table 3. **c**, Days to flowering after different periods of cold treatment (vernalization). Data from individual lines are grouped according to the STRUCTURE assignment in figure 4b. Admixed lines Arn1 and Mon3 omitted. **d**, Days to flowering grouped according to flowering class. **e**, Bidimensional plotting based on Non-metric Multidimensional Scaling (NMDS) analysis (scaling factors S1 and S2) grouping lines based Days to flowering. Samples are colored based on membership to STRUCTURE groups. Whiskers in the above plots extend to the most extreme data point which is no more than 1.5 times the IQR.

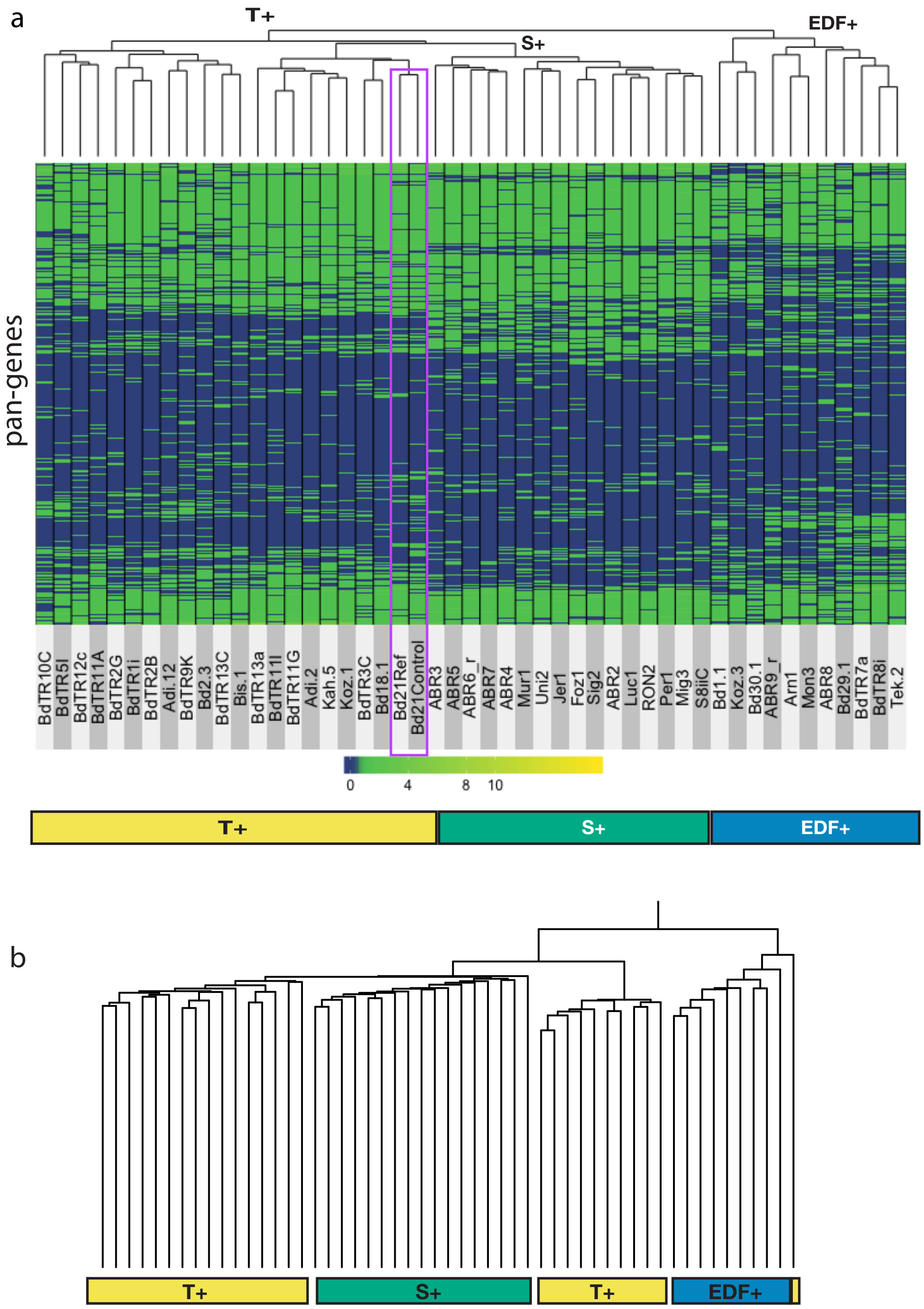

Supplementary Figure 7. Copy number and average nucleotide identity recover similar patterns of relatedness as the >3 million SNP-based ML tree. **a**, Lines grouped based on copy numbers for shell pan-genes. Copy number mapping to color scale is show below the plot. **b**, Lines grouped based on average nucleotide identities. Both approaches recovered similar major groupings of lines as STRUCTURE and the >3 million SNP-based ML tree (EDF+, T+ and S+).

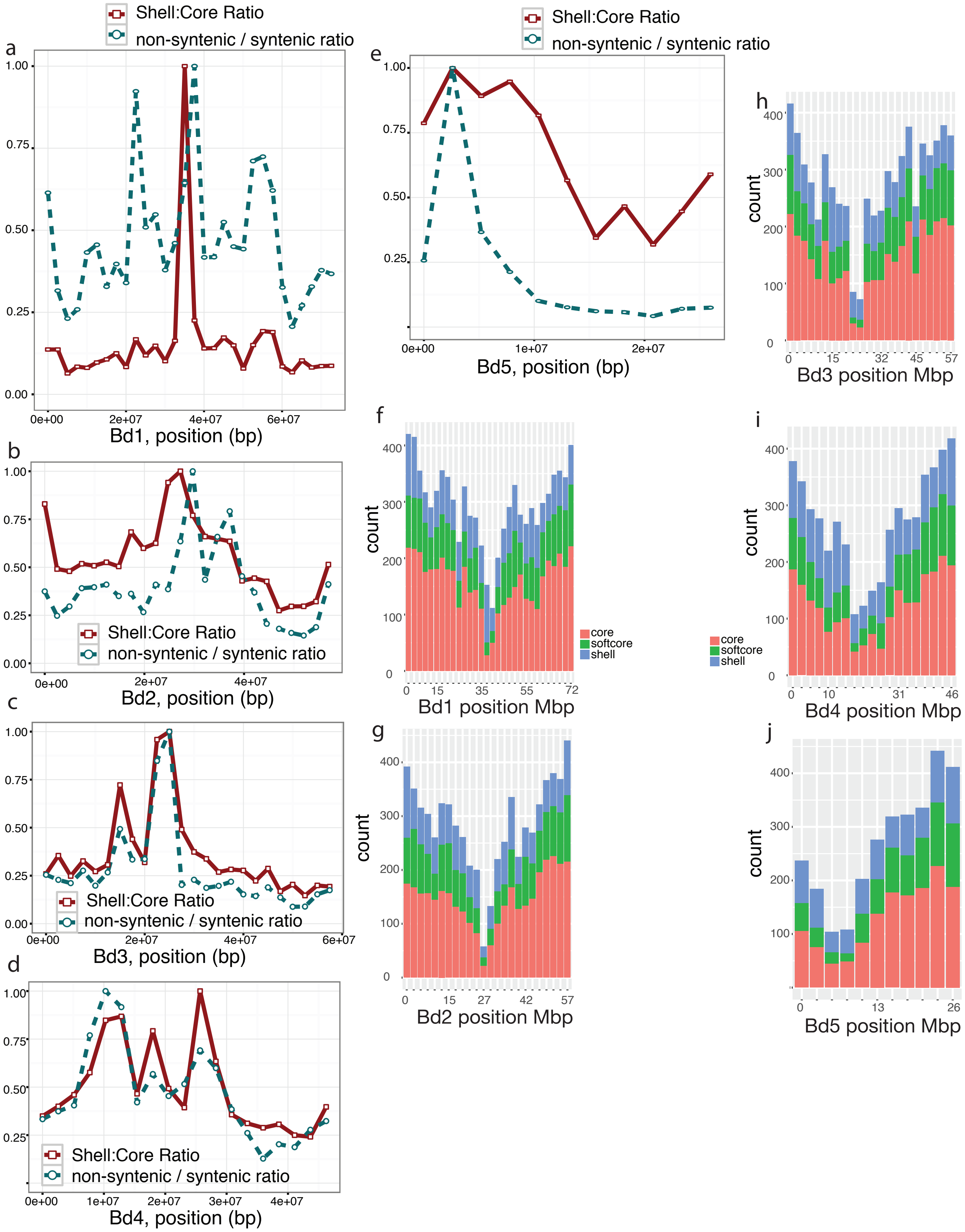

Supplementary Figure 8. Ratio of shell genes to core genes is closely correlated with the ratio of non-syntenic to syntenic genes in comparisons of the *B. distachyon* reference genome to the rice genome. Plots of chromosomes Bd1 (**a**), Bd2 (**b**), Bd3 (**c**), Bd4 (**d**). Bd5 (**e**). Shell genes are observed throughout the genome, but the ratio of shell genes to core genes is higher in pericentromeric regions of the chromosomes. Plots of core, soft-core and shell genes along chromosomes 1 (Bd1, **f**), 2 (Bd2, **g**), 3 (Bd3, **h**), 4 (Bd4, **i**) and 5 (Bd5, **j**).

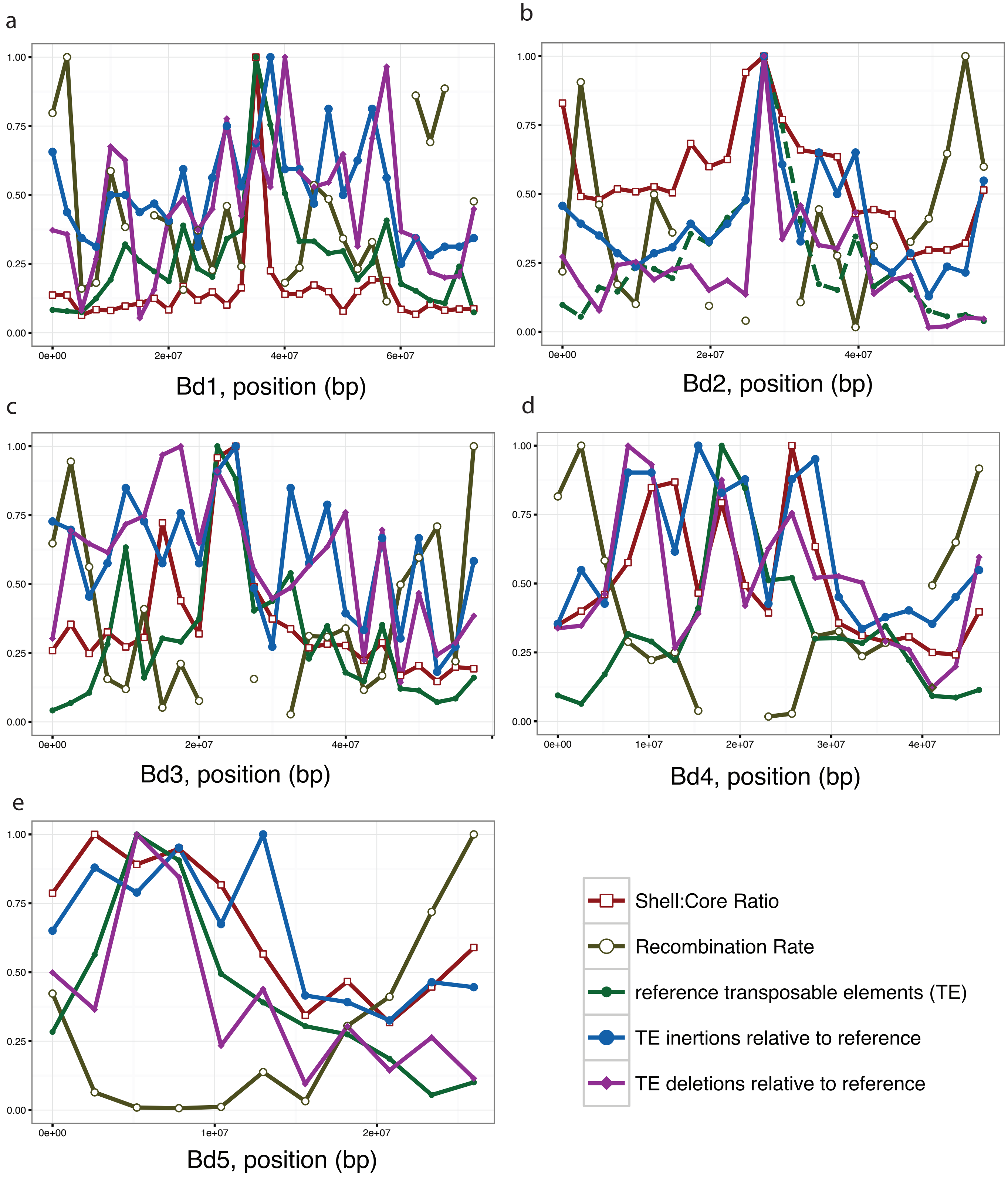

Supplementary Figure 9. a-e, Ratio of shell genes to core genes is higher in pericentromeric regions of the chromosomes, which have lower recombination rates, higher concentrations of annotated reference TEs, as well as higher frequency of TE insertion and deletion, relative to the reference genome of *B. distachyon*. Plots along chromosomes Bd1 (**a**), Bd2 (**b**), Bd3 (**c**), Bd4 (**d**). Bd5 (**e**). Y-values are scaled for each respective feature to the maximum value for that feature. Data points represent approximately 2.5 Mb bins, adjusted according to chromosome size. TE values are in percent coverage of the interval. Recombination rates are from Huo et al. 2011 Theor. and Appl. Genet. 123: 455-464 after mapping data to the version 2 reference genome assembly.

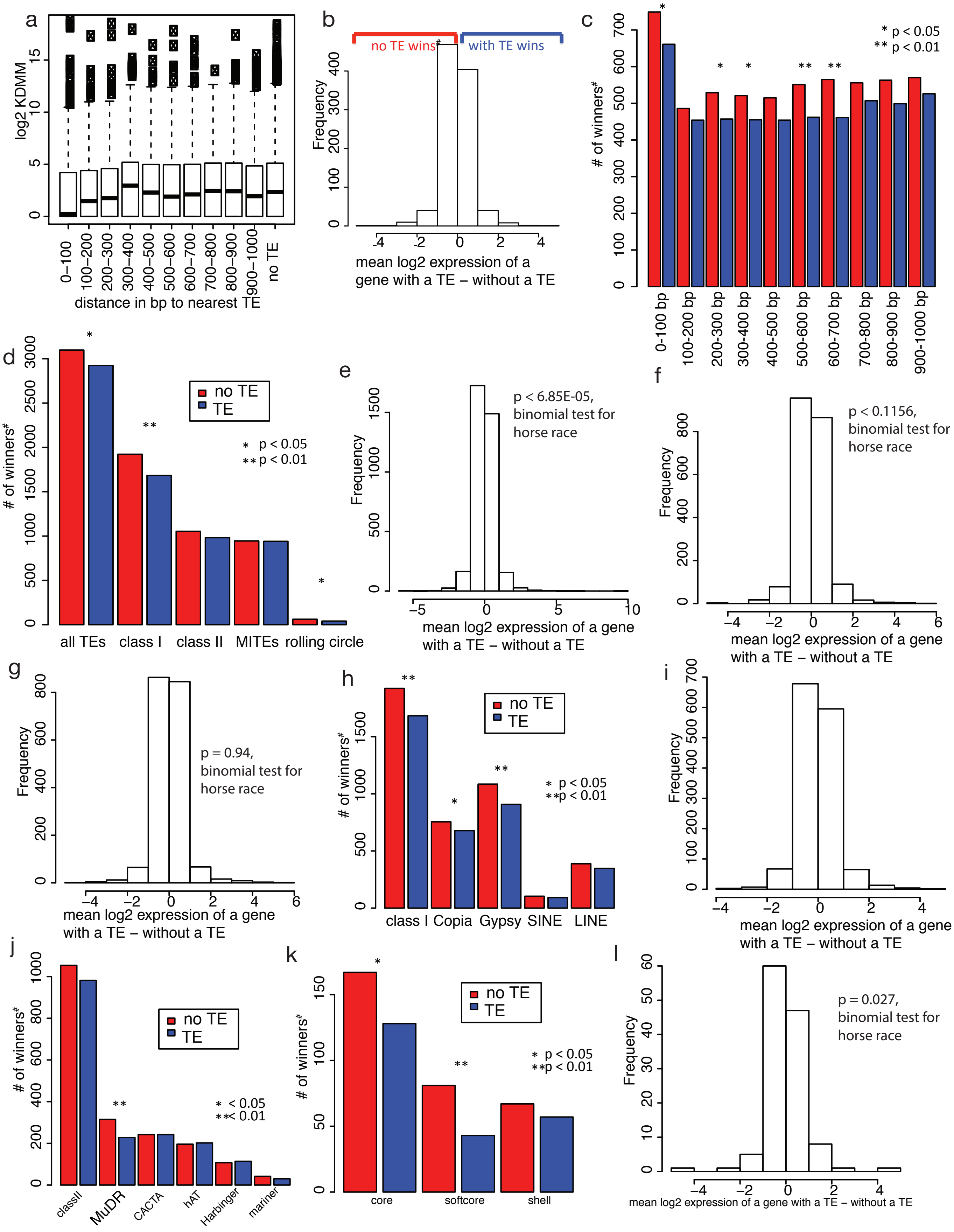
a
log2 KDMM
0–100
100–200
200–300
300–400
400–500
500–600
600–700
700–800
800–900
900–1000
no TE
distance in bp to nearest TE
b
no TE wins#
with TE wins
Frequency
mean log2 expression of a
gene with a TE – without a TE
c
# of winners#
* p < 0.05
** p < 0.01
0-100 bp
100-200 bp
200-300 bp
300-400 bp
400-500 bp
500-600 bp
600-700 bp
700-800 bp
800-900 bp
900-1000 bp
d
# of winners#
no TE
TE
* p < 0.05
** p < 0.01
all TEs
class I
class II
MITEs
rolling circle
e
Frequency
p < 6.85E-05,
binomial test for
horse race
mean log2 expression of a gene
with a TE – without a TE
f
Frequency
p < 0.1156,
binomial test for
horse race
mean log2 expression of a gene
with a TE – without a TE
g
Frequency
p = 0.94,
binomial test for
horse race
mean log2 expression of a gene
with a TE – without a TE
h
# of winners#
no TE
TE
* p < 0.05
**p < 0.01
class I
Copia
Gypsy
SINE
LINE
i
Frequency
mean log2 expression of a gene
with a TE – without a TE
j
# of winners#
no TE
TE
* < 0.05
**< 0.01
classII
MuDR
CACTA
hAT
Harbinger
mariner
k
# of winners#
no TE
TE
* p < 0.05
**p < 0.01
core
softcore
shell
l
Frequency
p = 0.027,
binomial test for
horse race
mean log2 expression of a gene with a TE – without a TE

Supplementary Figure 10. Among equivalent genes between lines that differ in the presence of an upstream TE, the version lacking a TE tends to be more highly expressed. **a**, Gene expression as a function of distance to upstream TE. **b**, Vertical axis plots the number of winners in horserace comparisons of syntenic genes amongst lines in which one gene has a flanking TE within 300-400 bp upstream and one does not. Horizontal axis plots expression difference between compared syntenic genes. **c**, Horserace results for different TE distance intervals. **d**, Effect of TE class on expression effect. **e-g**, Vertical axis plots the number of winners while horizontal axis plots expression difference between syntenic genes for class 1 TEs (**e**), class 2 TEs (**f**), and MITEs (**g**). **h**, Effect of different types of class 1 TEs on expression. **i**, Vertical axis plots the number of winners while horizontal axis plots expression difference between syntenic genes for Copia TEs. **j**, Number of winners in horserace comparisons, showing the effect of different types of class 2 TEs on expression. **k**, Number of winners in horserace comparisons, showing the effect of MuDR TE on expression of the different pan-genome subsets. **l**, Number of winners plotted over expression difference between the compared genes for MuDR TEs. Gene expression values are in KDMM-normalized counts; KDMM = kernel density mean of M component. Winner = higher expressed gene among lines.

Supplementary Table 1. List of *B. distachyon* accessions studied and their associated metadata.

Accession	Collection location	GOLD Biosample ID (JGI)	SRX accession	SRA sample	SRA study	Bioproject	Biosample	Elevation (meters)	Latitude	Longitude	Reference
ABR2	Hérault, France	Gb0017122	SRX182920	SRS360668	SRP001538	PRJNA32607	SAMN01162034	371	43° 36' 15.343" N	3° 15' 46.580" E	1
ABR3	Aísa, Huesca, Spain	Gb0017178	SRX2021046	SRS1615737	SRP081983	PRJNA337130	SAMN05518911	1928	42° 10' 49.8" N	0° 4' 23.2" W	1
ABR4	Arén, Huesca, Spain	Gb0017179	SRX2021047	SRS1615738	SRP081984	PRJNA337131	SAMN05519009	480	42° 15' 45.54" N	0° 43' 0.48" E	1
ABR5	Jaca, Huesca, Spain	Gb0017180	SRX182894	SRS360645	SRP001538	PRJNA32607	SAMN01161993	828	42° 34' 23.45" N	0° 33' 49.39" W	1
ABR6	Los Arcos, Navarra, Spain	Gb0017181	SRX298413	SRS438486	SRP001538	PRJNA32607	SAMN02194258	484	42° 34' 27.48" N	2° 11' 5.39" W	1
ABR7	Otero, Valladolid, Spain	Gb0017232	SRX2021065	SRS1615752	SRP081998	PRJNA337145	SAMN05519271	725	41° 35' 23.86" N	4° 45' 24.26" W	1
ABR8	Siena, Italy	Gb0017233	SRX874557<br>SRX874558	SRS844147	SRP054525	PRJNA258992	SAMN03003516	272	43° 18' 52.423" N	11° 19' 10.902" E	1
ABR9	Ljubjana, Croatia		SRX182919	SRS360667	SRP001538	PRJNA32607	SAMN01162033				1
Adi-2	Adiyaman, Turkey	Gb0017235	SRX2020496	SRS1615344	SRP081832	PRJNA337146	SAMN05519211	510	37° 46' 14.5" N	38° 21' 8.2" E	2
Adi10	Adiyaman, Turkey	Gb0009975	SRX185151	SRS361658	SRP001538	PRJNA32607	SAMN01163552	510	37° 46' 14.5" N	38° 21' 8.2" E	2
Adi-12	Adiyaman, Turkey	Gb0009864	SRX2020035	SRS1615304	SRP081813	PRJNA337348	SAMN05519184	510	37° 46' 14.5" N	38° 21' 8.2" E	2
Arn1	Arén, Huesca, Spain	Gb0009976	SRX298355	SRS438433	SRP024421	PRJNA249894	SAMN02194211	681	42° 15' 23.44" N	0° 43' 47.46" E	2
Bd1-1	Soma, Manisa, Turkey	Gp0001144	SRX060134<br>SRX060135<br>SRX060136<br>SRX116611	SRS190935	SRP001538	PRJNA32607	SAMN00262772	141	39° 11' 27.44" N	27° 36' 28.59" E	3
Bd2-3	Iraq	Gb0009943	SRX2020036	SRS1615305	SRP081814	PRJNA337363	SAMN05519268	42	33° 45' 39.18" N	44° 24' 11.07" E	3
Bd3-1	Iraq	Gp0001284	SRX117923	SRP001538	SRP001538	PRJNA32607	SAMN00779234	42	33° 45' 39.18" N	44° 24' 11.07" E	3
Bd18-1	Kaman, Kırşehir, Turkey	Gb0009918	SRX2020043,<br>SRX2020044	SRS1615310	SRP081819	PRJNA337356	SAMN05518944	1101	39° 22' 4.25" N	33° 43' 48.91" E	3
Bd21 Control	near Salakudin, Iraq	Gb0012676	SRX2020505,<br>SRX2020506	SRS1615350	SRP081838	PRJNA337420	SAMN05519133	42	33° 45' 39.18" N	44° 24' 11.07" E	3

Bd21-3	nar Salakudin, Iraq	Gp0039861	SRX119501 SRX146215	SRS291714 SRS312328	SRP010886	PRJNA250376	SAMN00788671 SAMN00991142	42	33° 45' 39.18" N	44° 24' 11.07" E	4
Bd29-1	Krym, Ukraine					PRJNA32607	SAMN07835970	260	44° 30' 55" N	33° 33' 23" E	3
Bd30-1	Dílar, Granada, Spain	Gp0001821	SRX116649 SRX059915	SRS190910	SRP001538	PRJNA32607	SAMN00262727	1220	36° 59' 25.76" N	3° 33' 31.44" W	5
BdTR1I	Aydin, Turkey	Gb0017239	SRX183383	SRS360859	SRP001538	PRJNA32607	SAMN01162198	841	38° 5' 35.03" N	28° 34' 59.02" E	6
BdTR2B	Turkey	Gb0012677	SRX2020498, SRX2020499	SRS1615346	SRP081834	PRJNA337421	SAMN05518874	667	40° 4' 55.55" N	31° 19' 52.01" E	6
BdTR2G	Ankara, Turkey	Gb0009917	SRX185148	SRS361655	SRP001538	PRJNA32607	SAMN01163549	1596	40° 23' 37.13" N	32° 59' 7.32" E	6
BdTR3C	Turkey	Gb0009942	SRX2020033	SRS1615302	SRP081811	PRJNA337362	SAMN05519130	1957	36° 46' 58.92" N	32° 57' 46.71" E	6
BdTR5I	Turkey	Gb0009974	SRX2020021	SRS1615296	SRP081805	PRJNA337382	SAMN05518814	1596	40° 23' 37.13" N	32° 59' 7.32" E	6
BdTR7A	Yozgat, Turkey	Gb0017240	SRX183377	SRS360854	SRP001538	PRJNA32607	SAMN01162193	1035	39° 44' 53.45" N	34° 39' 1.15" E	6
BdTR8I	Turkey	Gb0017241	SRX181206 SRX181207 SRX181208 SRX181209	SRS359840	SRP001538	PRJNA32607	SAMN01137370	2385	37° 6' 31.87" N	34° 4' 17.06" E	6
BdTR9K	Eskişehir, Turkey	Gb0009919	SRX2020011	SRS1615290	SRP081799	PRJNA337357	SAMN05518772	932	39° 45' 10.62" N	30° 47' 19.07" E	6
BdTR10C	Turkey	Gb0009946	SRX185149	SRS361656	SRP001538	PRJNA32607	SAMN01163550	1288	37° 46' 41.64" N	31° 53' 5.68" E	6
BdTR11A	Turkey	Gb0017236	SRX2020493	SRS1615342	SRP081830	PRJNA337147	SAMN05518768	986	38° 25' 0.42" N	28° 1' 52.75" E	6
BdTR11G	Kirklareli, Turkey	Gb0017237	SRX2020507	SRS1615351	SRP081839	PRJNA337148	SAMN05518884	124	41° 25' 17.86" N	27° 28' 36.81" E	6
BdTR11I	Turkey	Gb0009945	SRX2020031, SRX2020032	SRS1615301	SRP081810	PRJNA337374	SAMN05519029	363	39° 44' 17.39" N	28° 2' 24.71" E	6
BdTR12C	Turkey	Gp0009928	SRX059779 SRX059780	SRS190847	SRP001538	PRJNA32607	SAMN00262664	1035	39° 44' 53.45" N	34° 39' 1.15" E	6
BdTR13A	Ankara, Turkey	Gb0017238	SRX183318	SRS360828	SRP001538	PRJNA32607	SAMN01162158	787	39° 45' 23.35" N	32° 25' 56.46" E	6
BdTR13C	Ankara, Turkey	Gb0009863	SRX2020018	SRS1615294	SRP081803	PRJNA337347	SAMN05519253	1192	39° 24' 46.28" N	32° 59' 17.24" E	6

Bis1	Bismil, Turkey	Gp0017242	SRX2020040, SRX2020041, SRX2020042	SRS1615309	SRP081818	PRJNA337153	SAMN05518825	529	37° 52' 35.6" N	41° 0' 54.3" E	2
Foz1	Foz de Lumbier, Navarra, Spain	Gp0009893 (Mig1) Project ID: 404167	SRX2020038	SRS1615307	SRP081816	PRJNA337349	SAMN05518738	434	42º 38' 11.44" N	1º 18' 17.42" W	7
Gaz8	Gaziantep, Turkey	Gb0009947	SRX185147	SRS361654	SRP001538	PRJNA32607	SAMN01163548	891	37° 7' 39.8" N	37° 23' 26.9" E	2
Jer1	Ermita de San Jerónimo, Peraltilla, Huesca, Spain	Gp0009916 (Mon1) Project ID: 404166	SRX2020045	SRS1615311	SRP081820	PRJNA337354	SAMN05519060	418	42° 3' 16.56" N	0° 0' 44.57" W	7
Kah-1	Kahta, Turkey	Gp0017374	SRX2020494, SRX2020495	SRS1615343	SRP081831,	PRJNA337155	SAMN05519273	665	37° 44' 2.3" N	38° 32' 0.2" E	2
Kah-5	Kahta, Turkey	Gb0017182	SRX2020497	SRS1615345	SRP081833	PRJNA337133	SAMN05519229	665	37° 44' 2.3" N	38° 32' 0.2" E	2
Koz-1	Kozluk, Turkey	Gb0012678	SRX183517	SRS360986	SRP001538	PRJNA32607	SAMN01162329	853	38° 9' 8.2.6" N	41° 36' 34.8" E	2
Koz-3	Kozluk, Turkey	Gp0009991	SRX059781 SRX059782	SRS190848	SRP001538	PRJNA32607	SAMN00262665	853	38° 9' 8.2.6" N	41° 36' 34.8" E	2
Luc1	Ermita de Santa Lucía, Berdún, Huesca, Spain	Gp0017244	SRX1869528	SRS1520207	SRP076899	PRJNA249901	SAMN02821630	597	42° 36' 36.18" N	0° 53' 35.48" W	7
Mig3	San Miguel de Foces, Ibieca, Huesca, Spain	Gb0017183	SRX182705	SRS360564	SRP001538	PRJNA32607	SAMN01161923	572	42° 8' 52.76" N	0° 11' 41.89" W	7
Mon3	Puerto de Pallaruelo, Castejón de Monegros. Zaragoza, Spain	Gb0017184	SRX182916	SRS360665	SRP001538	PRJNA32607	SAMN01162031	515	41° 39' 4.75" N	0° 12' 37.51" W	7
Mur1	Castillo de Mur, Lleida, Spain	Gb0009944	SRX181229	SRS359860	SRP001538	PRJNA32607	SAMN01137389	487	42° 06' 18" N	0° 51' 23" E	7
Per1	Puerto del Perdón, Navarra, Spain	Gp0017243	SRX1869283	SRS1520047	SRP076878	PRJNA249907	SAMN02947395	742	42° 44' 13.34" N	1° 44' 58.6" W	7
Ron2	Roncal, Navarra, Spain	Gp0039823	SRX711596	SRS710321	SRP047542	PRJNA251304	SAMN02821496	594	42° 46' 50" N	0° 57' 48'' W	7

S8iiC	Zaidín,Huesca, Spain	Gb0017185	SRX2021637	SRS1616272	SRP082068	PRJNA337136	SAMN05519170	144	41° 36' 19.3" N	0° 08' 38.4" E	8
Sig2	Sigüés, Zaragoza, Spain	Gp0009900 (Sig1) Project ID: 404169	SRX2020046	SRS1615312	SRP081821	PRJNA337350	SAMN05518942	524	42° 36' 46.55" N	1° 0' 52.38" W	7
Tek-2	Tekirdag, Turkey	Gb0012679	SRX183516	SRS360985	SRP001538	PRJNA32607	SAMN01162328	20	41° 0' 40.1" N	27° 31' 8.8" E	2
Tek-4	Tekirdag, Turkey	Gb0017188	SRX2020048, SRX2020047	SRS1615313	SRP081822	PRJNA337137	SAMN05519158	20	41° 0' 40.1" N	27° 31' 8.8" E	2
Uni2	Escuela Politécnica Superior, Huesca, Spain	Gb0017189	SRX2021048	SRS1615739	SRP081985	PRJNA337138	SAMN05519210	480	42° 7' 3.98" N	0° 26' 42.81" W	7

Supplementary Table 2. Completeness Information for Individual Genome Assemblies.

inbred line	N50	CEGMA complete %	CEGMA partial %	primary transcripts	assembled genome size	BUSCO complete %
ABR2	46,617	94.76%	99.60%	32,811	270,545,830	97.5
ABR3	42,279	94.76%	99.60%	32,745	270,765,173	97.4
ABR4	49,653	94.35%	99.60%	32,389	267,583,885	97.2
ABR5	25,833	93.95%	99.60%	33,015	271,290,787	96.4
ABR6	38,433	95.16%	99.60%	32,449	283,715,612	97.9
ABR7	65,179	94.76%	99.60%	32,484	268,621,971	98.1
ABR8	NA	91.13%	97.98%	32,609	295,546,211	91.4
ABR9	57,868	95.16%	99.60%	29,649	273,963,599	96.8
Adi-10*	11,729	89.52%	98.39%	30,256	266,359,318	84.8
Adi-12	20,743	94.35%	99.19%	32,124	268,829,923	95.7
Adi-2	45,772	93.55%	99.19%	32,686	272,155,479	97.6
Arn1	84,176	95.97%	99.60%	32,269	267,544,414	97.9
Bd1-1	61,944	91.94%	97.18%	29,784	183,480,184	96.1
Bd18-1	1,027,801	95.56%	99.60%	32,403	269,291,862	98.4
Bd2-3	19,889	94.35%	99.60%	32,274	268,615,379	95.4
Bd21-3	162,708	95.16%	99.60%	31,970	243,777,234	96.7
Bd21Control	30,986	93.55%	98.79%	32,654	269,143,897	97.1
Bd29-1	14,197	92.34%	99.19%	31,613	253,064,284	93.6
Bd3-1*	80,194	89.52%	96.77%	25,845	170,261,895	85.6
Bd30-1	74,059	93.95%	98.79%	29,862	194,942,870	96.1
BdTR10C	15,470	93.55%	99.60%	31,500	268,286,720	93.2
BdTR11A	20,158	94.76%	98.79%	36,496	266,599,951	95.9
BdTR11G	45,335	95.16%	99.60%	32,867	272,988,239	97.6
BdTR11I	31,679	94.35%	99.60%	32,391	269,670,491	97.4
BdTR12c	15,138	93.95%	99.60%	31,960	247,594,554	94.8
BdTR13a	79,173	94.76%	99.60%	32,911	278,373,463	97.8
BdTR13C	18,488	93.15%	99.60%	32,282	267,610,339	95.1
BdTR1i	80,453	95.56%	99.60%	32,493	272,007,662	97.8
BdTR2B	28,026	94.76%	99.60%	32,239	269,783,561	96.7
BdTR2G	16,372	93.15%	98.79%	32,220	276,420,628	95.3
BdTR3C	417,778	95.56%	99.60%	32,336	263,477,856	97.9
BdTR5I	18,466	93.55%	99.19%	31,706	244,059,037	94.1
BdTR7a	46,933	94.35%	99.19%	32,266	270,408,172	97.3
BdTR8i	189,637	95.56%	98.79%	31,490	240,511,726	97.2
BdTR9K	15,934	93.95%	99.19%	32,193	269,356,293	94.6
Bis-1	24,103	94.35%	99.60%	32,559	269,200,960	96.9
Foz1	24,769	94.35%	99.19%	33,021	268,547,559	96.8
Gaz-8*	6,834	88.31%	98.39%	26,942	251,808,632	75
Jer1	22,190	94.76%	99.60%	32,793	264,870,977	96.5
Kah-1*	11,782	88.71%	97.98%	33,710	272,262,902	86.5
Kah-5	42,621	94.35%	99.19%	32,643	271,867,266	97.8

Koz-1	49,778	94.76%	99.60%	32,750	273,312,229	98.3
Koz-3	54,483	91.53%	97.18%	29,450	187,169,235	95.6
Luc1	69,053	94.76%	99.60%	32,340	264,910,967	97.9
Mig3	38,249	95.16%	99.60%	32,473	266,133,985	97.5
Mon3	29,829	94.76%	99.60%	32,655	269,626,778	96.6
Mur1	21,765	93.95%	99.19%	32,580	255,925,227	96.2
Per1	53,920	94.76%	99.60%	32,471	265,733,466	97.7
RON2	70,849	94.76%	99.60%	32,266	270,982,226	97.6
S8iiC	38,176	94.35%	99.19%	32,483	262,554,615	97.5
Sig2	21,539	94.76%	99.60%	33,075	268,379,077	96.8
Tek-2	27,391	94.35%	99.19%	31,701	239,806,349	96.7
Tek-4*	5,039	77.82%	94.76%	34,495	210,374,793	75.1
Uni2	19,865	94.35%	99.60%	32,441	268,147,538	97.5
version 1 sanger *B. distachyon* reference	426,629	95.56%	99.60%	25,532		
version 2 sanger *B. distachyon* reference	6,404,379	95.56%	99.60%	31,694		98.3
version 2 sanger *B. distachyon* reference, annotation control				32,712		98.1
Oropetium thomaeum (v1.0)						70.2
Carica papaya (ASGPB v0.4 Dec 2)						72
Brassica oleracea capitata (v1.0)						78.4
Arabidopsis halleri (v1.1)						85.9
Zea mays (v3.0)						92.2
Oryza sativa (MSU v7.0)						95.6
Setaria viridis (v1.1)						97.5
Sorghum bicolor (v3.1.1)						98.3

Setaria italica (v2.2)						98.4
Brachypodium stacei (v1.1)						98.6
Brachypodium distachyon (v3.1)						98.6
Oryza sativa Kitaake (v2.1)						98.8
Prunus persica (v2.1)						98.8
Arabidopsis thaliana columbia (TAIR 10)						99.3

* = not used in TE or non-coding analyses due to incomplete assembly

Supplementary Table 3. Percentage of membership of lines to each of the optimal K=3 genetic groups (EDF+, T+, S+) inferred from STRUCTURE analysis of >3 million SNP data. Accessions with significant admixed genetic profiles (showing percentage of membership <95% to its main population-group) are highlighted in bold.

Accession	EDF+	T+	S+
Bd1-1	1	0	0
Bd29-1	1	0	0
ABR9	1	0	0
BdTR7a	1	0	0
BdTR8i	1	0	0
Tek-2	1	0	0
Tek-4	1	0	0
**Arn1**	**0.819**	0	**0.181**
**Mon3**	**0.843**	0	**0.157**
**Bd21**	**0.155**	**0.845**	0
**Bd21-3**	**0.113**	**0.887**	0
**Bd2-3**	**0.07**	**0.93**	0
Bd3-1	0.044	0.95	0.006
Adi-2	0	1	0
Adi-10	0	0.983	0.017
Adi-12	0	1	0
Kah-1	0	1	0
Kah-5	0	1	0
Koz-1	0	1	0
Koz-3	0	0.964	0.036
Gaz-8	0	1	0
BdTR1i	0	1	0
BdTR2b	0	1	0
BdTR2g	0	1	0
BdTR3c	0	1	0
BdTR5i	0	1	0
BdTR9k	0	1	0
BdTR10c	0	0.956	0.044
BdTR11a	0	1	0
BdTR11g	0	1	0
BdTR11i	0	1	0
BdTR12c	0	0.984	0.016
BdTR13a	0	1	0
BdTR13c	0	1	0
**Bd18-1**	**0.052**	**0.819**	**0.129**
ABR2	0	0.002	0.998
ABR3	0	0	1
ABR4	0	0	1
ABR5	0	0	1
ABR6	0	0	1
ABR7	0	0.039	0.961
RON2	0	0	1
Per1	0	0	1
Luc1	0	0	1
Bd30-1	0	0.024	0.976
Jer1	0	0	1
Foz1	0	0	1
Sig2	0	0	1
Mur1	0	0	1
Bis-1	0	1	0
Mig3	0	0.003	0.997
Uni2	0.003	0.019	0.979
S8iiC	0	0	1

Supplementary Table 4. List of 25 flowering time traits, latitude and related molecular characters analyzed in 53 lines of *B. distachyon*. Trait number, trait abbreviation and description, number of trait states, and character states are indicated for each trait. All traits have been recorded as discrete (multistate) or binary characters. Traits Tr7-Tr25 correspond to polymorphisms in the flowering genes at the indicated positions. Color codes correspond to the colors used in Fig. 4c, d, e.

Trait number	Trait	Number of character states	Character states
Tr1	20hr NV (Days to flowering under 20hr days without vernalization)	5	A=120 (blue)<br>C=105-119 (red)<br>D=80-104 (Green)<br>E=49-79 (purple)<br>F=27-48 (orange)
Tr2	20hr 3V (Days to flowering under 20hr days after 3 weeks of vernalization)	6	A=120 (blue)<br>C=105-119 (yellow)<br>D=50-104 (red)<br>E=39-49 (orange)<br>F=25-38 (purple)<br>G=16-24 (green)
Tr3	20hr 6V (Days to flowering under 20hr days after 6 weeks of vernalization)	5	A=58-87 (blue)<br>C=30-57 (green)<br>D=25-29 (red)<br>E=20-24 (purple)<br>F=17-19 (orange)
Tr4	20hr 10V(Days to flowering under 20hr days after 10 weeks of vernalization)	4	A=58-71 (blue)<br>C=27-57 (red)<br>D=21-26 (green)<br>E=17-20 (orange)
Tr5	flowering class	6	1=ERF, extremely rapid flowering (yellow)<br>2=RF, rapid flowering (orange)<br>3=IRF, intermediate rapid flowering (purple)<br>4=IDF, intermediate delayed flowering (green)<br>5=DF, delayed flowering (red)<br>6=EDF, extremely delayed flowering (blue)
Tr6	Collection latitude	3	1 = >41.5 N (red)<br>2 = 34-41.5 N (blue)<br>3 = < 34 N (Green)<br>4= unknown (gray)
Tr7	PHYC_853	2	C 853 (red)<br>Y 853 (blue)
Tr8	PHYC_1114	2	I 1114 (red)

			T 1114 (blue)
Tr9	VRN2_186	2	E 186 (red)<br>G 186 (blue)
Tr10	FTL9_94	2	K 92 (red)<br>T 92 (blue)
Tr11	CO2_135	2	H 135 (red)<br>Y 135 (blue)
Tr12	CO2_152	2	1= single aa deletion (red)<br>0= no deletion (blue)
Tr13	CO2_217	2	A 217 (red)<br>T 217 (blue)
Tr14	CO2_235	2	F 235 (red)<br>S 235 (blue)
Tr15	CO2_250	2	G 250 (red)<br>S 250 (blue)
Tr16	FTL1_92	2	1= 80 aa deletion (red)<br>0= no deletion (blue)
Tr17	FTL13_7	2	I 7 (red)<br>V 7 (blue)
Tr18	FTL13_60	2	I 60 (red)<br>V 60 (blue)
Tr19	FUL2_19	2	G 19 (red)<br>V 19 (blue)
Tr20	FUL2_184del	4	1=deletion of aa 184-187 _4aa deletion (red)<br>2=deletion of aa 185-186 _2aa deletion (blue)<br>3=deletion of aa 186 _1aa deletion (orange)<br>0= no deletion (green)
Tr21	PPD1_273	2	L 273 (red)<br>R 273 (blue)
Tr22	PPD1_737	3	A 737 (red)<br>G 737 (green)<br>V 737 (blue)
Tr23	VRN1_intron1106	2	G (red)<br>T (blue)
Tr24	VRN1_intron1_4kb	2	1= 10bp deletion ~4kb intron1 (red)<br>0= no deletion (blue)
Tr25	VRN1_intron1_6kb	2	1= 9bp deletion ~6kb intron1 (red)<br>0= no deletion (blue)

Supplementary Table 5. Bayesian pairwise correlations of 19 binary flowering time traits, latitude and related flowering time molecular characters obtained for the dependent and independent models conducted in BayesTraits.

Traits	MCMC Dependent model harmonic mean (Lh)	MCMC Independent model harmonic mean (Lh)	LogBF	Correlation
5_6	-21,507	-20,017	-2,98	no (weak) evidence
5_7	-26,591	-32,522	11,862	very strong evidence
5_8	-26,291	-32,849	13,116	very strong evidence
5_9	-19,244	-27,329	16,17	very strong evidence
5_10	-30,193	-35,700	11,014	very strong evidence
5_11	-18,763	-22,174	6,822	strong evidence
5_12	-18,106	-21,705	7,198	strong evidence
5_13	-19,226	-22,494	6,536	strong evidence
5_14	-30,966	-36,620	11,308	strong evidence
5_15	-32,463	-33,763	2,600	positive evidence
5_16	-44,674	-44,007	-1,334	no (weak) evidence
5_17	-41,660	-42,978	2,636	positive evidence
5_18	-17,337	-25,623	16,572	very strong evidence
5_19	-37,951	-35,314	-5,274	no (weak) evidence
5_21	-25,831	-28,584	5,506	strong evidence
5_23	-33,014	-38,738	11,448	very strong evidence
5_24	-24,390	-32,280	15,780	very strong evidence
5_25	-24,983	-31,018	12,070	very strong evidence
6_7	-27,356	-27,122	-0,468	no (weak) evidence
6_8	-28,517	-25,442	-6,15	no (weak) evidence
6_9	-22,545	-19,681	-5,728	no (weak) evidence
6_10	-30,784	-28,947	-3,674	no (weak) evidence
6_11	-14,318	-13,778	-1,080	no (weak) evidence
6_12	-15,091	-14,164	-1,854	no (weak) evidence
6_13	-16,805	-15,089	-3,432	no (weak) evidence
6_14	-30,893	-30,394	-0,998	no (weak) evidence
6_15	-28,093	-26,155	-3,876	no (weak) evidence
6_16	-41,349	-37,551	-7,596	no (weak) evidence
6_17	-38,640	-36,619	-4,042	no (weak) evidence
6_18	-18,821	-18,015	-1,612	no (weak) evidence
6_19	-31,202	-28,522	-5,360	no (weak) evidence
6_21	-21,473	-22,400	1,854	no (weak) evidence
6_23	-34,558	-31,223	-6,670	no (weak) evidence
6_24	-26,633	-24,350	-4,566	no (weak) evidence
6_25	-26,653	-24,329	-4,648	no (weak) evidence
7_8	-23,955	-39,637	31,364	very strong evidence

7_9	-30,058	-32,716	5,316	strong evidence
7_10	-36,850	-41,710	9,72	strong evidence
7_11	-24,421	-26,561	4,28	positive evidence
7_12	-24,505	-26,559	4,108	positive evidence
7_13	-26,327	-27,515	2,376	positive evidence
7_14	-36,427	-42,084	11,314	very strong evidence
7_15	-37,055	-38,860	3,61	positive evidence
7_16	-50,029	-50,106	0,154	weak evidence
7_17	-45,018	-48,772	7,508	strong evidence
7_18	-25,357	-31,068	11,422	very strong evidence
7_19	-42,824	-41,321	-3,006	no (weak) evidence
7_21	-31,368	-33,467	4,198	positive evidence
7_23	-37,668	-44,937	14,538	very strong evidence
7_24	-29,872	-36,659	13,574	very strong evidence
7_25	-28,972	-36,221	14,498	very strong evidence
8_9	-28,045	-32,983	9,876	strong evidence
8_10	-37,683	-41,377	7,388	strong evidence
8_11	-24,808	-26,891	4,166	positive evidence
8_12	-24,973	-27,003	4,06	positive evidence
8_13	-26,201	-27,840	3,278	positive evidence
8_14	-34,841	-41,786	13,89	very strong evidence
8_15	-40,396	-38,698	-3,396	no (weak) evidence
8_16	-50,566	-50,541	-0,05	no (weak) evidence
8_17	-44,871	-48,676	7,61	strong evidence
8_18	-24,198	-31,328	14,26	very strong evidence
8_19	-42,494	-41,199	-2,59	no (weak) evidence
8_21	-31,885	-34,742	5,714	strong evidence
8_23	-37,261	-44,014	13,506	very strong evidence
8_24	-28,486	-36,299	15,626	very strong evidence
8_25	-28,350	-36,673	16,646	very strong evidence
9_10	-30,157	-35,538	10,762	very strong evidence
9_11	-21,767	-21,379	-0,776	no (weak) evidence
9_12	-19,592	-21,041	2,898	positive evidence
9_13	-18,278	-24,151	11,746	very strong evidence
9_14	-31,278	-36,051	9,546	strong evidence
9_15	-32,217	-32,602	0,77	weak evidence
9_16	-44,368	-44,323	-0,09	no (weak) evidence
9_17	-42,775	-42,802	0,054	weak evidence
9_18	-18,535	-24,804	12,538	very strong evidence
9_19	-36,868	-36,331	-1,074	no (weak) evidence
9_21	-27,046	-28,271	2,45	positive evidence

9_23	-34,392	-38,202	7,619	strong evidence
9_24	-25,868	-31,939	12,142	very strong evidence
9_25	-25,946	-30,817	9,742	strong evidence
10_11	-27,404	-30,842	6,876	strong evidence
10_12	-28,522	-28,493	-0,058	no (weak) evidence
10_13	-29,194	-30,555	2,722	positive evidence
10_14	-35,800	-45,125	18,65	very strong evidence
10_15	-38,008	-40,810	5,604	strong evidence
10_16	-53,404	-52,343	-2,122	no (weak) evidence
10_17	-50,099	-51,073	1,948	weak evidence
10_18	-27,130	-34,028	13,796	very strong evidence
10_19	-44,715	-44,028	-1,374	no (weak) evidence
10_21	-35,041	-36,488	2,894	positive evidence
10_23	-43,613	-46,714	6,202	strong evidence
10_24	-35,740	-38,983	6,486	strong evidence
10_25	-35,030	-39,749	9,438	strong evidence
11_12	-11,835	-14,621	5,572	strong evidence
11_13	-17,253	-16,266	-1,974	no (weak) evidence
11_14	-27,062	-31,774	9,424	strong evidence
11_15	-27,701	-26,682	-2,038	no (weak) evidence
11_16	-38,080	-37,604	-0,952	no (weak) evidence
11_17	-36,910	-39,153	4,486	positive evidence
11_18	-16,794	-19,255	4,922	positive evidence
11_19	-30,966	-29,922	-2,088	no (weak) evidence
11_21	-20,854	-22,434	3,16	positive evidence
11_23	-30,806	-33,910	6,208	strong evidence
11_24	-24,659	-24,499	-0,32	no (weak) evidence
11_25	-23,320	-25,218	3,796	positive evidence
12_13	-17,785	-16,379	-2,812	no (weak) evidence
12_14	-27,405	-31,793	8,776	strong evidence
12_15	-27,441	-28,533	2,184	positive evidence
12_16	-38,781	-38,591	-0,38	no (weak) evidence
12_17	-37,044	-37,345	0,602	weak evidence
12_18	-16,056	-18,039	3,966	positive evidence
12_19	-31,428	-29,481	-3,894	no (weak) evidence
12_21	-19,866	-21,972	4,212	positive evidence
12_23	-30,851	-32,472	3,242	positive evidence
12_24	-23,679	-24,731	2,104	positive evidence
12_25	-22,989	-24,199	2,42	positive evidence
13_14	-30,268	-31,154	1,772	weak evidence
13_15	-24,534	-28,984	8,9	strong evidence

13_16	-39,676	-39,054	-1,244	no (weak) evidence
13_17	-40,335	-37,891	-4,888	no (weak) evidence
13_18	-17,690	-19,326	3,272	positive evidence
13_19	-31,571	-30,096	-2,95	no (weak) evidence
13_21	-22,265	-23,123	1,716	weak evidence
13_23	-32,124	-34,479	4,71	positive evidence
13_24	-23,001	-26,103	6,204	strong evidence
13_25	-23,506	-25,704	4,396	positive evidence
14_15	-34,773	-42,535	15,524	very strong evidence
14_16	-52,315	-53,048	1,466	weak evidence
14_17	-49,974	-52,719	5,49	strong evidence
14_18	-27,654	-35,026	14,744	very strong evidence
14_19	-45,610	-44,194	-2,832	no (weak) evidence
14_21	-34,949	-41,428	12,958	very strong evidence
14_23	-43,953	-47,466	7,026	strong evidence
14_24	-35,683	-41,510	11,654	very strong evidence
14_25	-36,121	-39,809	7,376	strong evidence
15_16	-49,083	-49,279	0,392	weak evidence
15_17	-46,703	-50,272	7,138	strong evidence
15_18	-30,032	-30,466	0,868	weak evidence
15_19	-42,135	-40,598	-3,074	no (weak) evidence
15_21	-34,185	-33,671	-1,028	no (weak) evidence
15_23	-44,433	-43,317	-2,232	no (weak) evidence
15_24	-36,910	-36,229	-1,362	no (weak) evidence
15_25	-35,500	-37,013	3,026	positive evidence
16_17	-60,778	-59,713	-2,13	no (weak) evidence
16_18	-42,105	-41,988	-0,234	no (weak) evidence
16_19	-51,020	-52,282	2,524	positive evidence
16_21	-45,202	-45,256	0,108	weak evidence
16_23	-56,100	-54,898	-2,404	no (weak) evidence
16_24	-49,290	-47,704	-3,172	no (weak) evidence
16_25	-47,780	-47,924	0,288	weak evidence
17_18	-40,543	-40,858	0,63	weak evidence
17_19	-51,159	-52,593	2,868	positive evidence
17_21	-45,304	-44,695	-1,218	no (weak) evidence
17_23	-47,115	-54,040	13,85	very strong evidence
17_24	-45,896	-47,000	2,208	positive evidence
17_25	-46,044	-46,954	1,82	weak evidence
18_19	-35,592	-33,979	-3,226	no (weak) evidence
18_21	-22,565	-26,503	7,876	strong evidence
18_23	-32,183	-35,907	7,448	strong evidence

18_24	-22,594	-28,345	11,502	very strong evidence
18_25	-23,260	-28,306	10,092	very strong evidence
19_21	-37,141	-36,942	-0,398	no (weak) evidence
19_23	-47,448	-46,296	-2,304	no (weak) evidence
19_24	-40,799	-39,152	-3,294	no (weak) evidence
19_25	-40,473	-40,933	0,92	weak evidence
21_23	-37,533	-39,418	3,77	positive evidence
21_24	-28,373	-32,040	7,334	strong evidence
21_25	-30,178	-31,703	3,05	positive evidence
23_24	-36,345	-41,985	11,28	very strong evidence
23_25	-36,774	-41,996	10,444	very strong evidence
24_25	-22,832	-34,497	23,33	very strong evidence

Supplementary Table 6. Number of Leaves on parent shoot at time of flowering (Mean and SD "+/-"). Plants grown under 20 hours light/day. NV=no vernalization, 3wkV= 3 weeks of vernalization, 6wkV = 6 weeks of vernalization, and 10wkV = 10 weeks of vernalization. ERF=extermely rapid flowering, RF=rapid flowering, IRF=intermediate rapid flowering, IDF=intermediate delayed flowering, DF=delayed flowering, and EDF=extremely delayed flowering.

inbred line	NV	3wkV	6wkV	10wkV	Flowering Class
Bd21	6.0 +/- 0.00	4.2 +/- 0.408	5.0 +/- 0.00	4.5 +/- 0.548	ERF
Bd3-1	7.0 +/- 0.00	5.0 +/- 0.00	4.8 +/- 0.408	4.8 +/- 0.408	RF
Bd21-3	6.7 +/- 0.516	4.0 +/- 0.00	4.7 +/- 0.516	4.5 +/- 0.548	RF
Bd30-1	9.0 +/- 0.753	4.8 +/- 0.408	5.0 +/- 0.00	5.0 +/- 0.00	IRF
Bd2-3	10 +/- 1.03	5.0 +/- 0.00	5.0 +/- 0.00	5.0 +/- 0.00	IRF
BdTR13a	12 +/- 4.55	5.0 +/- 0.00	5.2 +/- 0.408	5.0 +/- 0.00	IRF
BdTR2B	12 +/- 2.48	4.5 +/- 0.548	5.0 +/- 0.00	5.0 +/- 0.00	IRF
BdTR13C	11 +/- 2.34	4.7 +/- 0.512	4.8 +/- 0.408	5.0 +/- 0.00	IRF
Bis-1	11 +/- 1.45	4.0 +/- 0.00	5.0 +/- 0.00	5.0 +/- 0.00	IRF
BdTR11I	12 +/- 1.55	5.4 +/- 0.890	5.0 +/- 0.00	5.0 +/- 0.00	IDF
Arn1	13 +/- 0.707	4.2 +/- 0.408	4.6 +/- 0.548	5.0 +/- 0.00	IDF
Mon3	14 +/- 1.82	5.0 +/- 0.00	5.0 +/- 0.00	5.0 +/- 0.00	IDF
Gaz-8	13 +/- 0.987	5.1 +/- .628	5.0 +/- 0.00	5.0 +/- 0.00	IDF
BdTR5I	15 +/- 0.577	5.3 +/- 0.456	5.0 +/- 0.00	5.0 +/- 0.00	IDF
BdTR2G	15 +/- 1.79	4.4 +/- 0.548	5.0 +/- 0.00	5.0 +/- 0.00	IDF
BdTR3C	16 +/- 1.12	5.0 +/- 0.00	5.0 +/- 0.00	5.0 +/- 0.00	IDF
Adi-12	17 +/- 0.945	4.3 +/- 0.578	4.5 +/- 0.707	5.0 +/- 0.00	IDF
BdTR10C	16 +/- 0.949	5.0 +/- 0.00	5.0 +/- 0.00	4.5 +/- 0.707	IDF
Kah-1	16 +/- 3.16	4.7 +/- 0.516	5.0 +/- 0.00	5.0 +/- 0.00	IDF
BdTR1i	16 +/- 2.33	4.2 +/- 0.448	5.0 +/- 0.00	5.0 +/- 0.00	IDF
Koz-1	18 +/- 0.782	4.0 +/- 0.00	5.0 +/- 0.00	5.0 +/- 0.00	IDF
Adi-2	18 +/- 1.67	4.8 +/- 0.667	5.0 +/- 0.00	5.0 +/- 0.00	IDF
Kah-5	19 +/- 2.03	4.0 +/- 0.00	5.0 +/- 0.00	5.0 +/- 0.00	IDF
BdTR11G	17 +/- 1.26	5.5 +/- 0.577	5.0 +/- 0.00	5.0 +/- 0.00	IDF
BdTR11A	17 +/- 1.50	5.5 +/- 0.577	5.0 +/- 0.00	5.0 +/- 0.00	IDF
BdTR9K	17 +/- 0.916	4.2 +/- 0.402	5.0 +/- 0.00	4.0 +/- 0.00	IDF
Adi-10	17 +/- 1.22	4.6 +/- 0.812	4.8 +/- 0.402	5.0 +/- 0.00	IDF
Koz-3	19 +/- 1.02	5.0 +/- 0.00	5.0 +/- 0.00	5.0 +/- 0.00	IDF
BdTR12c	17 +/- 0.753	5.8 +/- 0.408	5.0 +/- 0.00	4.8 +/- 0.406	IDF
Per1	18 +/- 2.09	6.7 +/- 0.812	5.7 +/- 0.516	5.5 +/- 0.548	IDF
Uni2	>19 +/- 0.00	6.2 +/- 1.20	5.0 +/- 0.00	5.5 +/- 0.548	DF
ABR3	>19 +/- 0.00	7.0 +/- 0.578	5.5 +/- 0.578	5.0 +/- 0.00	DF
ABR8	18 +/- 2.35	6.5 +/- 1.65	5.5 +/- 0.578	5.0 +/- 0.00	DF
ABR7	>19 +/- 0.00	6.7 +/- 0.812	4.7 +/- 0.516	5.0 +/- 0.00	DF
Bd18-1	>19 +/- 0.00	6.8 +/- 1.22	5.0 +/- 0.00	5.5 +/- 0.548	DF
Mig3	>19 +/- 0.00	6.2 +/- 1.56	5.2 +/- 0.216	5.0 +/- 0.00	DF
Bd1-1	>19 +/- 0.00	7.0 +/- 0.894	4.8 +/- 0.401	5.0 +/- 0.00	DF
RON2	>19 +/- 0.00	7.0 +/- 0.354	6.0 +/- 0.408	6.5 +/- 0.546	DF
ABR5	>19 +/- 0.00	9.1 +/- 1.80	6.3 +/- 0.500	6.8 +/- 0.673	DF
ABR4	>19 +/- 0.00	7.5 +/- 0.837	8.0 +/- 1.22	6.0 +/- 0.00	DF
Mur1	>19 +/- 0.00	7.0 +/- 0.00	5.0 +/- 0.00	6.1 +/- 1.22	DF
ABR6	>19 +/- 0.00	8.2 +/- 1.17	5.8 +/- 0.401	5.0 +/- 0.00	DF
Sig2	>19 +/- 0.00	8.3 +/- 3.67	5.8 +/- 0.753	5.0 +/- 0.00	DF
Luc1	>19 +/- 0.00	8.5 +/- 1.64	6.0 +/- 0.00	5.7 +/- 0.517	DF
ABR2	>19 +/- 0.00	10 +/- 1.23	5.0 +/- 0.00	5.0 +/- 0.00	DF
Foz1	>19 +/- 0.00	8.7 +/- 2.54	6.1 +/- 0.412	5.0 +/- 0.00	DF
BdTR7a	>19 +/- 0.00	16 +/- 3.45	13 +/- 1.94	12 +/- 0.894	EDF
Tek-2	>19 +/- 0.00	18* +/- 4.56	16 +/- 0.983	13 +/- 2.48	EDF
BdTR8i	>19 +/- 0.00	>19 +/- 0.00	16 +/- 1.17	16 +/- 1.02	EDF
Tek-4	>19 +/- 0.00	>19 +/- 0.00	16 +/- 0.983	15 +/- 1.43	EDF
Bd29-1	>19 +/- 0.00	>19 +/- 0.00	16 +/- 3.51	6.0 +/- 0.516	EDF
ABR9	>19 +/- 0.00	9.5 +/- 1.74	8.0 +/- 0.629	7.0 +/- 1.21	EDF

Supplementary Table 7. Days to Heading (Mean and SD "+/-"). Plants grown under 20 hours light/day. NV=no vernalization, 3wkV= 3 weeks of vernalization, 6wkV = 6 weeks of vernalization, and 10wkV = 10 weeks of vernalization. ERF=extermely rapid flowering, RF=rapid flowering, IRF=intermediate rapid flowering, IDF=intermediate delayed flowering, DF=delayed flowering, and EDF=extremely delayed flowering.

	NV	3wkV	6wkV	10wkV	Flowering Class
Bd21	27 +/- 0.837	16 +/- 0.516	17 +/- 0.00	17 +/- 0.00	ERF
Bd3-1	33 +/- 3.09	19 +/- 1.03	20 +/- 0.632	18 +/- 0.837	RF
Bd21-3	32 +/- 3.27	16 +/- 0.00	17 +/- 0.00	17 +/- 0.00	RF
Bd30-1	49 +/- 6.09	20 +/- 0.408	21 +/- 0.00	20 +/- 0.00	IRF
Bd2-3	51 +/- 6.87	21 +/- 4.55	20 +/- 0.408	22 +/- 0.577	IRF
BdTR13a	68 +/- 9.79	23 +/- 0.707	22 +/- 0.816	21 +/- 0.447	IRF
BdTR2B	63 +/- 20.89	21 +/- 3.33	23 +/- 0.548	27 +/- 2.22	IRF
BdTR13C	63 +/- 5.31	23 +/- 1.55	22 +/- 1.09	21 +/- 0.00	IRF
Bis-1	67 +/- 7.63	20 +/- 1.03	28 +/- 16.4	22 +/- 1.34	IRF
BdTR11I	73 +/- 24.8	28 +/- 2.54	25 +/- 1.33	24 +/- 1.03	IDF
Arn1	79 +/- 11.1	25 +/- 3.14	25 +/- 0.516	24 +/- 0.00	IDF
Mon3	79 +/- 13.2	26 +/- 2.5	23 +/- 1.55	22 +/- 2.04	IDF
Gaz-8	84 +/- 29.5	20 +/- 1.10	21 +/- 0.00	21 +/- 0.00	IDF
BdTR5I	89 +/- 5.77	28 +/- 2.61	27 +/- 5.13	24 +/- 0.00	IDF
BdTR2G	83 +/- 8.64	22 +/- 3.2	22 +/- 1.16	26 +/- 2.12	IDF
BdTR3C	87 +/- 4.45	22 +/- 1.73	21 +/- 0.00	22 +/- 0.894	IDF
Adi-12	99 +/- 11.3	21 +/- 0.00	20 +/- 2.00	22 +/- 1.95	IDF
BdTR10C	93 +/- 14.3	25 +/- 1.86	25 +/- 3.40	28 +/- 0.00	IDF
Kah-1	93 +/- 14.9	21 +/- 1.03	21 +/- 0.00	21 +/- 0.00	IDF
BdTR1i	100 +/- 6.78	22 +/- 2.95	22 +/- 0.50	24 +/- 0.00	IDF
Koz-1	103 +/- 9.91	23 +/- 2.58	21 +/- 2.51	17 +/- 0.408	IDF
Adi-2	105* +/- 11.3	20 +/- 0.5	23 +/- 0.816	26 +/- 0.983	IDF
Kah-5	105 +/- 3.71	20 +/- 0.00	21 +/- 1.15	24 +/- 0.50	IDF
BdTR11G	103 +/- 9.9	25 +/- 2.87	24 +/- 0.957	21 +/- 0.00	IDF
BdTR11A	108* +/- 9.79	29 +/- 2.00	24 +/- 0.577	20 +/- 2.12	IDF
BdTR9K	103 +/- 9.29	21 +/- 1.09	20 +/- 0.812	21 +/- 0.00	IDF
Adi-10	101 +/- 9.98	22 +/- 1.33	20 +/- 0.00	21 +/- 0.00	IDF
Koz-3	113* +/- 5.64	26 +/- 1.83	26 +/- 2.30	27 +/- 1.38	IDF
BdTR12c	113 +/- 8.21	30 +/- 2.32	22 +/- 1.03	23 +/- 1.83	IDF
Per1	106* +/- 21.1	36 +/- 3.34	27 +/- 1.75	29 +/- 2.25	IDF
Uni2	>120 +/- 0.00	34 +/- 13.1	25 +/- .408	22 +/- 2.25	DF
ABR3	>120 +/- 0.00	35 +/- 3.46	28 +/- 0.00	25 +/- .564	DF
ABR8	>120 +/- 0.00	35 +/- 5.16	25 +/- 1.02	23 +/- .768	DF
ABR7	110* +/- 13.3	36 +/- 5.72	23 +/- 1.96	27 +/- 2.21	DF
Bd18-1	>120 +/- 0.00	41 +/- 6.99	25 +/- 0.00	28 +/- 0.00	DF
Mig3	115* +/- 23.7	45 +/- 18.8	25 +/- 1.47	27 +/- 2.22	DF
Bd1-1	>120 +/- 0.00	47 +/- 8.17	24 +/- 1.22	25 +/- 2.00	DF
RON2	>120 +/- 0.00	46 +/- 7.50	36 +/- 4.55	25 +/- 2.21	DF
ABR5	>120 +/- 0.00	47 +/- 12.0	35 +/- 2.38	38 +/- 1.50	DF
ABR4	>120 +/- 0.00	50 +/- 5.33	42 +/- 6.71	33 +/- 1.15	DF
Mur1	111* +/- 5.23	51 +/- 21.4	26 +/- 1.86	30 +/- 1.51	DF
ABR6	>120 +/- 0.00	52 +/- 10.3	32 +/- 2.00	35 +/- 3.00	DF
Sig2	119* +/- 10.3	52 +/- 14.1	30 +/- 2.64	30 +/- 3.21	DF
Luc1	118* +/- 0.00	62 +/- 11.2	27 +/- 1.79	32 +/- 3.29	DF
ABR2	>120 +/- 0.00	64 +/- 11.5	22 +/- .983	27 +/- 2.12	DF
Foz1	>120 +/- 0.00	74 +/- 16.8	27 +/- 3.02	31 +/- 2.74	DF
BdTR7a	>120 +/- 0.00	105* +/- 24.5	68 +/- 11.46	63 +/- 7.15	EDF
Tek-2	>120 +/- 0.00	>120 +/- 0.00	87 +/- 10.2	71 +/- 9.79	EDF
BdTR8i	>120 +/- 0.00	111* +/- 20.1	79 +/- 10.9	65 +/- 4.35	EDF
Tek-4	>120 +/- 0.00	>120 +/- 0.00	80 +/- 6.33	58 +/- 5.50	EDF
Bd29-1	>120 +/- 0.00	>120 +/- 0.00	91* +/- 14.3	27 +/- 3.14	EDF
ABR9	>120 +/- 0.00	65 +/- 21.4	58 +/- 20.4	28 +/- .577	EDF

*asterisk denotes some plants within treatment did not flower after 120 days of growth

>greater than sign denotes plants did not flower after 120 days of growth

Extremely Rapid Flowering (ERF), Rapid Flowering (RF), Intermediate Rapid Flowering (IRF) Intermediate Delayed Flowering (IDF), Delayed Flowering (DF) and Extremely Delayed Flowering (EDF)

Supplementary Notes

Supplementary Note 1. *De novo* genome assembly of 54 inbred lines

We assessed the accuracy of the assemblies by alignment of raw reads to respective genomes using BWA and SAMtools and analyzing read depth and coverage as well as the presence of nucleotide variants. We generated three different Bd21 (reference genome line) short-read data sets for this study. In all three datasets, 99.99% of the Bd21 reference genome was covered by sequence alignments, leaving only 8,965 bp; 6,396 bp; and 5,922 bp covered by fewer than three reference short-reads, respectively. Alignment of Bd21 short-reads to 51 non-reference genome assemblies yielded a median of 1 Mbp of genic sequence (Fig. 1c) and greater than 8 Mbp of non-genic sequence (~3% of the genome in total) with <3 aligned reference short-reads, indicating that these sequences were missing or highly diverged from the reference accession. The control of mapping non-reference reads from two respective lines to their *de novo* assemblies produced only 234 kbp and 405 kbp of read coverage of <3, whereas reference short-reads (Bd21) validated the presence of hundreds of genes absent from the reference line (Supplementary Fig. 2c).

Similar to mapping reference reads to our non-reference assemblies, mapping reads from the non-reference accessions onto the reference genome yielded a median of 1.5 Mb of genic sequence (3,067 non-redundant reference genes in total) and greater than 7 Mb of non-genic sequence with read coverage <3 (Fig. 1c). Importantly, deleted and highly divergent regions in our assemblies are distributed throughout genome, as shown by the frequency of gene deletion relative to the reference (Supplementary Fig. 2). Thus, by aligning short-reads, we verified that numerous regions in the assembled genomes are not found in the reference genome and that many of these novel sequences are found in multiple accessions.

We constructed a pan-genome based on whole-genome (WG) sequences by iteratively scanning each of the 54 genome assemblies and adding DNA sequences that were > 600 bp long (enough to contain a small gene) and did not contain any 21 bp sequence found in the preceding sequences using Vmatch with the qnomatch option (http://www.vmatch.de). The average size of the DNA blocks not contained in the reference genome was ~1.5 kb. In total, 57,402 blocks were over 1 kb, and these sequences were added to the reference sequence to produce a 391 Mb WG pan-genome. Scaffold sequences were covered by contigs over 99.3% of their total length.

Supplementary Note 2. Correlated evolution of flowering time traits and related molecular traits

Correlated evolution of traits was investigated using BAYESTRAITS[9]. We analyzed 19 (out of 25) traits, representing the 5 flowering-time traits (1-5) by a single trait (flowering class, 5) to avoid redundancy. Flowering traits (Tr1-Tr4) and flowering class trait (Tr5) are set according to Ream et. al. (2014)[10] and Woods et al. (2016)[11]. For simplicity, flowering class and latitude were re-coded as binary traits (flowering class: EDF *vs* non-EDF; latitude: <34°N *vs* >34°N) and were correlated to 17 binary gene traits. The program computes pairwise correlation analysis between binary traits, allowing to test for correlated evolution by comparing the fit (log-likelihood) of two continuous-time Markov models, the independent model in which the two traits evolve independently on the tree, and the dependent model in which the traits evolve in a correlated fashion such that the rate of change in one trait depends upon the background state of the other. We calculated the logarithm of the harmonic mean of the likelihoods for each model through Bayesian MCMC searches of 1010000 iterations, with 10000 burn in and sampling every 1000 iterations. We applied the Bayes Factor (BF) Test statistic (Log BF = 2(log[harmonic mean(dependent model)] – log[harmonic mean(independent model)]) for which values <2 indicate weak evidence, 2-5 positive evidence, 5-10 strong evidence and >10 very strong evidence of correlation between any pair of traits (Supplementary Table 5).

BF tests for potential correlated evolution of 19 (out of 25) binary flowering time traits and genes on the pruned SNP tree (Fig. 4a, g-j, Supplementary Figs. 5, 6a) indicated a strong (BF values =5-10) and very strong (BF >10) evidence of correlation for 13 traits (7-14, 18, 21, 23-25) with flowering class (trait 5) and to each other, and positive evidence (BFT=2-5) for two other traits (15, 17) (Supplementary Table 5). Conversely, all 18 examined traits showed weak evidence (BF <2) of correlated evolution with latitude (Supplementary Table 5; Supplementary Fig. 6b). These results support flowering class and its associated flowering time traits and genes as the main factors driving intra-specific divergence within wild populations of *B. distachyon* and discard the influence of latitudinal distribution on it.

We observed strong population structure in which lines fell into three major groups that correlated first with flowering behavior and secondly with geographic origin: a group containing mostly late-flowering lines from a broad geographic area, and a group containing earlier flowering lines, separated into two eastern and western geographical subgroups (Fig. 4a-f). Most of the lines that make up the extremely delayed flowering (EDF+) group required extended vernalization, ≥6 weeks (Supplementary Fig. 6c-e, Supplementary Tables 6 and 7). The western S+ lines group consists largely of lines that require long vernalization, around 6 weeks of cold (Fig. 4c, traits 1-5). In contrast, all the lines within the eastern T+ group (including the reference line Bd21) require 3 weeks or less of vernalization (Fig. 4c traits 1-5; Supplementary Table 6,7 for details). The three main clades can in part be recapitulated in NMDS clustering of the extensive flowering data provided in this study, with the exception of the admixed Mon3 and Arn1 (Supplementary Table 3 and 7, Supplementary Fig. 6e). Lines Mon3 and Arn1 in the EDF group showed an admixture of roughly 16-18% from the western group and these

lines flower earlier than the other EDF lines (Fig. 4c traits 1-5; Supplementary Tables 3 and 7, Supplementary Fig. 6c-e).

We found strong correlations between flowering behavior and variants within several flowering time genes (particularly the EDF+ clade), which may contribute to the different flowering behaviors and provide testable hypotheses for future studies (SNPs and indels in coding and non-coding regions of *PHYC*, *VRN1*, *VRN2*, *FTL1*, *FTL9*, *FTL13*, *FUL2*, *CO2*, *PPD1*; traits 6-14, 16-18, 20-25; Supplementary Table 5; Supplementary Fig. 6b).

Supplementary Note 3. Influence of intra-species TE activity on shell:core ratio by linear model analysis

In addition to the stringent TE filtering during annotation, we evaluated the number of TEs potentially miss-annotated as genes by performing a BLASTN search using all primary CDSs annotated in *B. distachyon* as a query against the TREP database. Genes for which more than 75% of the sequence was covered by a TE with more than 95% of sequence identity were considered as potential miss-annotated genes. Only six genes were identified as putative TEs or TE derived sequences, ruling out an effect of miss-annotation on the GLM analysis or the effect of TEs on gene expression.

The potential impact of TE variants on the gene shell:core ratio was then tested using Generalized Linear Models (GLM, logit link). In our models, the shell:core ratio calculated in 2.5Mbp fixed intervals (provided as a number of successes, number of failures) was entered as a dependent variable and the ratio of TE non-reference insertions:core insertions or the ratio of absent insertions:core insertions were entered as explanatory variables. Models were run in R 3.1.3. Pseudo McFadden R^2 were calculated in R using the library (BaylorEdPsych). The shell:core ratio was significantly influenced by the intra-species TE activity (non reference insertion and deletion) relative to the reference line ($z= 19.4$, $df= 107$, $p<2e-16$, pseudo $R^2=0.42$ and $z= 17.6$, $df= 107$, $p<2e-16$,pseudo $R^2=0.35$ respectively). For each model, the normal distribution of the residuals were confirmed with QQplots.

In order to investigate the respective contribution of the shell/core ratio and TE activity on the loss of synteny, we first built a full glm model where the non-syntenic/syntenic ratio (provided as a number of successes, number of failures) was entered as a dependent variable and the shell/core ratio, non-reference TE, deletion TE and all their interactions were entered as predictors. Interactions, however, were dropped as non-significant. We then compared the percentage of deviance explained by the full model (without interaction) to the percentage of deviance explained by successive models from which we either dropped the shell/core ratio, the non-reference TE or the deletion TE from the predictors.

Supplementary Methods

De novo genome assembly of 54 inbred lines

We assembled 54 genomes for divergent *B. distachyon* inbred lines, including a reference control. Five genome assemblies (Gaz-8, Kah-1, Tek-4, Bd3-1, Adi-10) were smaller than expected, probably because they were assembled from HiSeq2000 data with shorter read lengths (75 bp) and lower sequencing depth. Since the amount of genic sequence contained in the lower-quality assemblies was similar to the average and reference genome, we included them in the construction of the pan-genome, but excluded them from analyses for which complete non-coding sequence was required.

While the total lengths of our assemblies were about the same as the reference genome, even the best assemblies were in many smaller pieces (range: 957-73,776 scaffolds). To make the assemblies easier to work with and provide physical context, for each assembly we used synteny to the reference genome to order and orientate scaffolds into five pseudomolecules corresponding to the five *B. distachyon* chromosomes and an additional super-scaffold containing unassigned sequence. Synteny was identified with CoGe SynMap[12] (https://genomevolution.org/CoGe/SynMap.pl) using the default parameters with a minimal number of genes set to two for the *Brachypodium distachyon* Bd21(v2.0)[13] genome. We used the CoGe SynMap syntenic path assembly algorithm to order scaffolds into syntenic pseudomolecules based on the inferred synteny to the reference, in which scaffolds were joined using 100 "N"s. For the best assembly, 97% of the total sequence was placed into five chromosomes[12] (Fig. 1b). On average 72% of the total assembly length of each line was placed into five pseudomolecules. Scaffolds that were too small or contained too few genes to be positioned via synteny were concatenated, separated by Ns, and included in the final assembly as an accessory sequence. Significantly, no reference sequence was added to any of the assemblies during this process. Syntenically ordered scaffolds provided the chromosomal location for novel sequence that was contained on scaffolds with flanking syntenic anchors. Thus, utilizing high-quality whole-genome assemblies and syntenic information is sufficient to identify the physical position of most novel sequences.

Final assemblies were evaluated for errors and corrected using the alignments of the raw reads to the individual assemblies using BWA[14] (BWA v.0.7.10) and SAMtools (http://www.htslib.org) (SAMtools v. 0.1.18). BUSCO (v2)[15] as well as CEGMA[16] were used to assess the completion of the genome assemblies based on the presence of core eukaryotic genes (Supplementary Fig. 1, Supplementary Table 2). The software was run with default parameters with the included reference dataset.

Gene annotation

Annotation began by creating assembled transcripts for three *B. distachyon* lines (Bd21, Bd21-3, Bd1-1) for which we had deep Illumina paired-end RNA-seq transcriptome data. For Bd1-1, 57,070 transcript assemblies were constructed using PASA (v. r2011_05_20)[17] from 58,292 RNA-seq transcripts derived from Bd1-1 assemblies

using PERTRAN (JGI plant reference-based transcriptome assembly pipeline). For Bd21, 72,305 transcript assemblies were constructed using PASA from 310,090 ESTs derived from PERTRAN and 454 sequences. For Bd21-3, 56,184 transcript assemblies were constructed using PASA from 59,927 RNA-seq transcripts derived from Bd21-3 paired-end Illumina RNA-seq reads using PERTRAN pipeline.

In the general annotation pipeline, loci were determined by alignments of the assembled transcripts above and/or EXONERATE (v. 2.2.0)[18] alignments of proteins from six plants (*Arabidopsis thaliana, Glycine max, Sorghum bicolor, Oryza sativa, Setaria italic* and *Vitis vinifera*) and the Swiss-Prot database (uniprot eukaryote 07/2014) of eukaryote proteins to the respective soft-repeat masked genome using RepeatMasker[19] with up to 2K BP extension on both ends unless extending into another locus on the same strand. Gene models were predicted by homology-based predictors, FGENESH+ (v. 2.6)[20], FGENESH_EST (similar to FGENESH+, EST as splice site and intron input instead of protein/translated ORF), and GenomeScan[21].

The highest scoring predictions for each locus were selected using multiple positive factors including EST and protein support, and one negative factor: overlap with repeats. The selected gene predictions were improved by PASA. Improvement includes adding UTRs, splicing correction, and adding alternative transcripts. PASA-improved gene model proteins were subject to protein homology analysis to the above mentioned proteomes to obtain Cscore and protein coverage. Cscore is a protein BLASTP score ratio to MBH (mutual best hit) BLASTP score, and protein coverage is the highest percentage of protein aligned to the best of homologs. PASA-improved transcripts were selected based on Cscore, protein coverage, EST coverage, and CDS overlap with repeats. Transcripts were selected if they had a Cscore ≥0.5 and protein coverage ≥0.5, or if they had EST coverage and less than 20% of the CDS overlaped repeats. For gene models where >20% of the CDS overlapped repeats, its Cscore must be at least 0.9 and homology coverage at least 70% to be selected. The selected gene models were subject to Pfam analysis and gene models whose protein is more than 30% in Pfam TE domains were removed.

Bd21, Bd21-3, Bd1-1 were annotated via the above annotation pipeline to produce a set of high-confidence full-length transcripts. Annotation yielded 30,683 primary transcript models for Bd1-1, 30,995 primary transcript models for Bd21-3 and 31,532 primary transcript models for Bd21. Transcripts from these three annotated genomes were filtered for gene model completeness and transcriptome or high homology support to proteomes from other species. The filtered transcripts were further filtered for any sign of TE protein domains or sequence redundancy (473,807 sequences). These filtered models were then used as full-length transcripts in annotating all 55 genome assemblies (including two reference controls), as well as the WG pan-genome compiled from unannotated DNA sequence using Vmatch. The WG pan-genome annotation identified 45,863 primary loci, of which 38,390 were deemed complete by the presence of a valid start and stop codon. As a control, we re-annotated the reference genome with the same pipeline, yielding 32,712 primary loci, of which 30,877 were complete.

Gene Ontology enrichment

Genes were functionally annotated using PFAM, KOG, and Panther using the primary transcript. Interproscan was used to assign GO terms. Enrichment of Gene Ontology terms was determined by topGO (v. 2.22)[22] and additionally with goatools[23] (v0.6.10) in order to obtain FDR estimates for particular enrichments.

Transposable element annotation and analysis for individual *B. distachyon* lines

Transposable elements (TEs) and repeats were annotated in additional detail for the following *B. distachyon* lines in order to determine their effect on adjacent gene expression: ABR5, ABR3, ABR6, ABR4, ABR2, ABR7, ABR8, Bd18-1, BdTR2g, BdTR2b, BdTR1i, BdTR3d, Koz-1, Bis-1, BdTR13a, BdTR11g, BdTR11i, BdTR10c, BdTR5i, Kah-5, BdTR12c, BdTR9k, Adi-12, Adi-2, Bd2-3, Bd21 and ABR9. RepeatMasker[19] (v. 4-0.5) was run on *B. distachyon* genomes using RMBlast and a combined library of exemplar repetitive DNA elements (oxyrep.ref, grasrep.ref, mcotrep.ref) downloaded from RepBase20.06 (http://www.girinst.org/repbase/).

TEs were assigned to the class of the best matching repetitive element from the repeat library. Genes from the cloud pan-genome were removed prior to downstream analysis. For each of the 27 *B. distachyon* lines listed above, BedTools[24] was used to map repetitive elements to the 1 kb region upstream from the translation start site of the gene model with the longest transcript and to determine repetitive element coverage in the same region. Expression levels (KDMM-normalized counts) were log2-transformed and values ≤1 were set to 0. Syntenic orthologs across the above genome assemblies were determined by aliasing genes to their reference genome ortholog, using an in-house lift-over pipeline (described below) that uses synteny information, as described below. Statistical analyses and plots were performed using R.

Identification of homeologous gene pairs in Fig. 5f

Homeologous gene pairs were identified by running CoGe SynMap[12] (https://genomevolution.org/CoGe/SynMap.pl) and filtered using Quota Align at a 1:1 syntenic depth on hard-masked *Brachypodium distachyon* Bd21(v2.0)[13] against itself. SynMap outputs a list of syntenic blocks of genes along with percentage nucleotide identity. Genes from blocks with 60-87% nucleotide identity were retained as homeologous gene pairs from the pre-grass whole-genome duplication.

Leaf expression data for 36 inbred lines

To estimate transcript abundance for each gene model, we used transcriptome sequencing of greenhouse grown plants from 36 accessions. To estimate transcript abundance for each gene model, we used transcriptome sequencing of greenhouse grown plants from 36 accessions. Seeds of each accession were sowed in 600mL of moist Profile porous ceramic rooting media (Profile Products) in Deepot D40H pots and then cold-stratified at 6°C for 14d to ensure synchronized germination. Two replicates of each accession were

grown for 21days at 25°C and we then harvested the two youngest, fully-expanded leaves of each plant directly onto liquid nitrogen. RNA was extracted with the Spectrum Plant Total RNA kit (Sigma) according to the manufacturers specifications. We also performed an on-column DNase treatment (Qiagen). We prepared short-read RNASeq libraries using a 3' tag-seq approach as described[25]. Raw reads were quality filtered and mapped using two different approaches.

For quantifying expression of cloud genes, we mapped filtered reads to respective pan-gene transcripts using BWA mem, processed these using picard, and sorted reads were then stacked and counted by gene model using each genome's cognate gene annotation.

For plots in Figure 3e and the Transposable Element analysis (see below, gene expression levels across 27 assemblies to evaluate the effect of TEs on gene expression), we mapped filtered reads to the reference Bd21 v2.1 genome (downloaded from Phytozome on 9 April 2014) using BWA[14]. Mapping files were processed using picard tools (http://broadinstitute.github.io/picard) and then stacked and counted by gene model (Bd21 v2.1 annotation). Sequence tags from each library were normalized to sequencing depth using the KDMM procedure in JMPGenomics 6.1 (SAS Institute).

Expression analysis of *B. distachyon* interaction with wheat stem rust and diurnal sampling

We performed an RNAseq experiment on the interaction of the non-reference accession, Bd1-1, with the grass fungal pathogen *P. graminis* f. sp. *tritici* (*Pg-tr*), a pathogen of wheat and barley at two different time points. Three independent replicates were performed for both mock treatment and *Pg-tr* treatment. For all trials sampling of 12 and 18 hpi were performed at 6 am and 11:30 am, respectively. This corresponded to dawn and midday, allowing us to also observe genes that are under diurnal regulation. Leaves were collected and immediately flash frozen in liquid nitrogen before storage at -80°C. Frozen leaves were ground in liquid nitrogen using a mortar and pestle. Total RNA extractions were performed using Concert™ Plant RNA Reagent (Invitrogen). mRNA was prepared into a library using ScriptSeq™ v2 RNA-Seq Library Preparation Kit (Epicentre) following the stranded (dUTP) protocol. 101bp single-end Illumina sequencing was performed, generating raw reads. Raw fastq file reads were filtered and trimmed using the JGI QC pipeline resulting in the filtered fastq and raw reads were evaluated for artifact sequence by kmer matching (kmer=25), allowing 1 mismatch and detected artifact was trimmed from the 3' end of the reads. RNA spike-in reads, PhiX reads and reads containing any Ns were removed. Quality trimming was performed using the phred trimming method set at Q6. Filtered reads from each library were aligned to the reference genome using HISAT[26] (v. 0.1.4-beta) and featureCounts[27] was used to generate the raw gene counts. DESeq2[28] (v. 1.10.0) was subsequently used to determine which genes were differentially expressed between pairs of conditions. The parameters used to call a gene DE between conditions were p-value < 0.05.

B. distachyon lineage-specific genes and expression breadth of genes

Data on genes specific to *B. distachyon* (lineage-specific, 1 × 1) versus shared relative to rice and sorghum was taken from a remapping of gene identifiers to the v2.1 *B. distachyon* annotation based on published orthoMCL comparisons to the three species[29]. Gene expression data (Fragments Per Kilobase of transcript per Million mapped reads, FPKM) for nine distinct tissues, representing a wide-range of tissue types of the *B. distachyon* Bd21 reference line was used for determining expression breadth[29]. In our study, narrow gene expression was defined as expression in fewer than or equal to 3 tissues types, while broad expression was defined as expression in 7 or more tissue types. KDMM-normalized expression data for 36 accessions was plotted for core versus shell genes, present within the reference annotation, in Fig. 3e.

Clustering pan-gene models

As annotated genes residing on each individual assembly were not necessarily syntenic, we identified related genes across assemblies by grouping the 1,796,495 gene models across assemblies by sequence similarity using Markov clustering in the GET_HOMOLOGUES-EST pipeline[30] (https://github.com/eead-csic-compbio/get_homologues) with minimum alignment coverage of 75%. This algorithm takes BLASTN hits, adding up all non-overlapping aligned segments and computing coverage over the shortest sequence, to drive Markov clustering of CDS sequences. This strategy was found to be more robust to partial gene models and skipped exons than CD-HIT-EST. This resulted in 61,155 pan-genome clusters. The 61,155 pan-genome clusters were reduced to 57,819 non-redundant gene clusters by collapsing clusters with ≥95% identity and ≥75% alignment coverage. We then selected a single gene from each cluster as the pan-gene representative for further analysis. For clusters containing a gene from the v2.1 reference annotation the reference gene was selected as the pan-gene. For clusters without a reference gene the gene from the first line in the cluster (alphabetical) was selected as the pan-gene (correspondence between pan-genes and genes within clusters is provided through phytozome: https://phytozome.jgi.doe.gov/pz/portal.html). To validate the presence/absence variation inferred from the transcript clustering, raw reads were aligned to the pan-genes (genomic sequence) using BWA (BWA v. 0.7) and SAMtools (SAMtools v. 0.1.18). Genes with a read depth <3 over more than 20% of the gene were considered absent.

dN/dS calculation

CDS nucleotide sequences and corresponding peptide sequences of pan-genome single-copy clusters with occupancy ≥ 4 (the minimum number of sequences required for these calculations) were selected. For each cluster, peptide sequences were aligned with clustal-omega[31] (v1.2.1) and the resulting alignments translated back to codon-based nucleotide alignments using the primers4clades suite[32]. Each DNA alignment was then passed to yn00_cds_prealigned, obtained from[33] (https://github.com/hyphaltip/subopt-kaks), to estimate the ratio of nonsynonymous substitutions per nonsynonymous site (dN) to the number of synonymous substitutions per synonymous site (dS) of all pairs of

sequences in a cluster. Pairs with with dS=0 were assigned omega=0. The resulting plot was produced with R (https://www.r-project.org) *boxplot* function and parameters notch=T, varwidth=F, outline=F.

Pan-genome size simulations

These simulations were performed with GET_HOMOLOGUES-EST[30], the same software used to cluster pan-genome sequences (https://github.com/eead-csic-compbio/get_homologues), with parameters -M –c. The simulations estimate how many novel CDS nucleotide sequences are added when the set of 56 *B.distachyon* genomes are iteratively sampled in random order. For each genome, CDS sequences with BLASTN multi-hsp coverage ≥ 75% and sequence identity ≥90% to previously processed CDS are considered homologues and thus not added to the growing pan-genome pool. Sequences internally labelled as inparalogues are also discarded. Note that the order in which genomes are added affects the estimated pool size, and for this reason sampling was replicated 20 times.

Transfer non-reference gene models to the reference genome

In stepwise fashion we attempted to transfer non-reference gene models onto the reference genome using our in-house liftover pipeline. First, each non-reference locus' genomic sequence including introns, if any, and up to 1 kb of upstream and downstream sequence is obtained. For intergenic spaces less than 2 kb, the sequence is split evenly between the two adjacent loci. The non-reference locus sequences were then mapped to the Bd21 v2.0 reference genome using BLAT. Sequences mapped to more than one location were assigned to a unique location based on the synteny of neighboring genes. When a locus genomic sequence mapped to the reference genome uniquely and 100%, the gene model is perfectly transferred to the new genome. For the remaining gene models, both their transcript and CDS sequences were mapped with BLAT[24] to a region in the reference genome as described mapping above. Gene models were then made from CDS alignments with ≥95% identity over ≥90% of the CDS, valid splice sites if any, and were considered transferred if the resulting peptide was ≥70% similar to the peptide predicted from the original gene model. UTRs were added, if any, using transcript alignments. Still untransferred gene models were mapped to the reference genome using GMAP (v. 2007-09-28)[34] Gene models based on GMAP alignments with quality of 95% identity, 70% coverage and valid splice sites if any are transferred if and only if resulting gene model peptide is 70% or more similar to the original gene model peptides and in a location not occupied by gene models transferred in earlier steps.

For gene models that were not transferred under the criteria of 70% or greater peptide similarity to the original model, we masked the *B. distachyon* reference genome for annotated genes and subsequently used exonerate[18] to scan the presence of gene remnants.

Phylogenetic analysis

For whole genome phylogenetic analysis, maximum Likelihood (ML) phylogenetic analysis was performed on the high-confidence SNPs of the 53 *B. distachyon* lines in RAxML[35] (v. 8.0.0). We imposed the General Time Reversible model with gamma distribution and a proportion of invariant sites (GTR+I+G) substitution model in the searches. This model was selected as the optimal model after a pilot study with a reduced data set by jMODELTEST (v. 2) based on the Akaike Information Criterion (AIC)[36, 37]. We computed 20 ML starting trees from 20 alternative randomized Maximum Parsimony (MP) trees, searching for best-scoring ML trees and estimating branch support for the best tree from 1000 bootstrap replicates (BS). RAxML trees were mid-point rooted. The best ML tree with bipartitions bootstrap support of branches was visualized with FigTree (v. 1.4.0) (http://tree.bio.ed.ac.uk/software/figtree/).

Genomic structure among the *B. distachyon* lines was assessed with STRUCTURE (v. 2.3.4)[38], imposing an admixture ancestry model and a correlated allele frequencies model. We estimated values of genomic-group differentiation (K) between 1 and 6, considering that up to 3 main genetic groups were detected in our phylogenetic ML analysis (see Fig. 4a). Each search consisted of an initial burn-in of 5000 Markov Chain Monte Carlo (MCMC) iterations followed by 5000 additional MCMC iterations, and estimation of cluster membership (q) set to a 10% threshold value. Ten replicates were run for each K. The number of genomic groups (clusters) in the data was estimated using STRUCTURE HARVESTER (v. 0.9.94)[39], which identifies the optimal *K* based both on the posterior probability of the data for a given K and the rate of change in the likelihood distribution among Ks (ΔK; Evanno et al. 2005). These outcomes were visualized using the software DISTRUCT (v. 1.1)[40].

F_{ST} between the three major groups identified by STRUCTURE was estimated by vcftools[41] using the weighted Weir and Cockerham method on the SNP data file.

Inbreeding coefficient of each line was estimated by vcftools[41] using the 3,933,264 SNP data set. The median inbreeding coefficient was 0.8846 which is consistent with the normally inbreeding nature of *B. distachyon* and the inbreeding of most lines in the lab. The reference line, Bd21, which has undergone seven generations of single seed descent, had a coefficient of 0.9988, indicating that it is nearly entirely homozygous. Line Uni2 was an outlier, having a coefficient of 0.4848 suggesting that is was recently hybridized.

Phylogenetic analysis of *VRN1*

For the pan-genome *BdVRN1* tree, the Bd21 *VRN1* sequence (including the putative 1.5kb promoter, exons and introns, total of 10.76kb) was used to perform a BLASTN search in the assembled genomes of 48 lines (Adi-10, Bd3-1, Gaz-8, Tek-4 and ABR8 were not included). In 46 accessions the BLASTN search identified one unambiguous hit in each genome and the corresponding sequences were extracted. In the remaining two accessions, Kah-1 and ABR5, the 10.76kb sequence had to be manually assembled

because the sequence was split across two contigs. The 49 sequences (including the Bd21 control) were aligned using MAFFT and a phylogenetic tree was constructed using PHYML with 100 bootstrap replicates, both performed in Geneious (v 9.1.5) (http://www.geneious.com)[42].

For the read mapping *BdVRN1* tree, the variants of the different accessions (in VCF format) were implemented on the reference sequence using a custom script in Geneious, resulting in 47 sequences corresponding to the genomic sequences for the 47 lines as predicted by the read mapping variants. These sequences, along with the Bd21 control were aligned using MAFFT and a phylogenetic tree was constructed using PHYML with 100 bootstrap replicates in Geneious.

Flowering time measurements

Growth chamber temperatures averaged 21°C during the 16-hour light period and 18°C during the 8-hour dark period. Flowering time of vernalized plants was measured as the number of days from the end of vernalization (or emergence of the coleoptile in non-vernalized plants) to the first day upon which emergence of the spike was detected. The number of primary leaves derived from the parent culm was recorded at the time of heading to control for development[43].

Analysis of flowering time traits and related molecular traits evolution

We looked at variants in known vernalization and flowering time genes that influence flowering responsiveness in *B. distachyon.* We identified and analyzed specific mutations and indels in coding and non-coding regions of candidate vernalization and flowering time genes (*PHYC, VRN1, VRN2, FTL1, FTL9, FTL13, FUL2, CO2, PPD1*) (traits 6-14, 16-18, 20-25; Supplementary Table 4)[43]. The evolution of the 25 flowering time traits and related molecular traits recorded in the 53 sequenced *B. distachyon* lines was analyzed using BAYESTRAITS v 2.0[9]. Because our ML tree was fully resolved (Supplementary Fig. 4), we estimated ancestral state reconstruction of the 25 discrete flowering time characters on this phylogeny, in which the admixed lines Arn1 and Mon3 were removed using the drop.it option of the R package APE[44], leaving a tree of 49 tips and 48 nodes. Ancestral character state reconstruction was performed for each trait through Maximum Likelihood (ML) searches of 1000 iterations for multistate/binary characters. The inferred probabilities of ancestral states at tree nodes were mapped onto the tree using the plot option of APE (Fig. 4, Supplementary Fig. 5 and 6).

Genome-wide feature plots and analysis

BEDTools was used to identify intersects between indels and other features as well as calculate feature frequency and/or coverage among genomic windows[45]. Plots and correlations were produced with R (https://www.r-project.org) using the ggplot2 library. Recombination rates are from published work[46]. We mapped the coordinates from the version 1 genome to the version 2 assembly for our calculations.

Supplementary References

1. Draper J*, et al. Brachypodium distachyon*. A new model system for functional genomics in grasses. *Plant Physiology* **127**, 1539-1555 (2001).

2. Vogel JP, Tuna M, Budak H, Huo N, Gu YQ, Steinwand MA. Development of SSR markers and analysis of diversity in Turkish populations of *Brachypodium distachyon*. *BMC Plant Biology* **9**, Art. No.: 88 (2009).

3. Vogel JP, Garvin DF, Leong OM, Hayden DM. *Agrobacterium*-mediated transformation and inbred line development in the model grass *Brachypodium distachyon*. *Plant Cell Tiss Org Cult* **85**, 199-211 (2006).

4. Vogel J, Hill T. High-efficiency *Agrobacterium*-mediated transformation of *Brachypodium distachyon* inbred line Bd21-3. *Plant Cell Reports* **27**, 471-478 (2008).

5. Gordon SP*, et al.* Genome diversity in Brachypodium distachyon: Deep sequencing of highly diverse inbred lines. *Plant Journal* **79**, 361-374 (2014).

6. Filiz E, Ozdemir BS, Budak F, Vogel JP, Tuna M, Budak H. Molecular, morphological, and cytological analysis of diverse *Brachypodium distachyon* inbred lines. *Genome* **52**, 876-890 (2009).

7. Mur LAJ*, et al.* Exploiting the Brachypodium Tool Box in cereal and grass research. *New Phytologist* **191**, 334-347 (2011).

8. Rabe F*, et al.* A complete toolset for the study of Ustilago bromivora and Brachypodium sp. as a fungal-temperate grass pathosystem. *Elife* **5**, (2016).

9. Meade A, M. P. BayesTraits v 2.0. . (2014).

10. Ream TS*, et al.* Interaction of photoperiod and vernalization determines flowering time of Brachypodium distachyon. *Plant Physiology* **164**, 694-709 (2014).

11. Woods DP, McKeown MA, Dong Y, Preston JC, Amasino RM. Evolution of VRN2/Ghd7-like genes in vernalization-mediated repression of grass flowering. *Plant Physiology* **170**, 2124-2135 (2016).

12. Tang H, Lyons E, Pedersen B, Schnable JC, Paterson AH, Freeling M. Screening synteny blocks in pairwise genome comparisons through integer programming. *BMC Bioinformatics* **12**, 102 (2011).

13. Goodstein DM*, et al.* Phytozome: a comparative platform for green plant genomics. *Nucleic Acids Res* **40**, D1178-1186 (2012).

14. Li H, Durbin R. Fast and accurate short read alignment with Burrows-Wheeler transform. *Bioinformatics* **25**, 1754-1760 (2009).

15. Simao FA, Waterhouse RM, Ioannidis P, Kriventseva EV, Zdobnov EM. BUSCO: assessing genome assembly and annotation completeness with single-copy orthologs. *Bioinformatics* **31**, 3210-3212 (2015).

16. Parra G, Bradnam K, Korf I. CEGMA: a pipeline to accurately annotate core genes in eukaryotic genomes. *Bioinformatics* **23**, 1061-1067 (2007).

17. Haas BJ*, et al.* Improving the Arabidopsis genome annotation using maximal transcript alignment assemblies. *Nucleic Acids Res* **31**, 5654-5666 (2003).

18. Slater GS, Birney E. Automated generation of heuristics for biological sequence comparison. *BMC Bioinformatics* **6**, 31 (2005).

19. Smit AFA, Hubley R, Green P. RepeatMasker Open-3.0., (1996-2011).

20. Salamov AA, Solovyev VV. Ab initio gene finding in Drosophila genomic DNA. *Genome research* **10**, 516-522 (2000).

21. Yeh RF, Lim LP, Burge CB. Computational inference of homologous gene structures in the human genome. *Genome Res* **11**, 803-816 (2001).

22. Alexa A, Rahnenfuhrer J, Lengauer T. Improved scoring of functional groups from gene expression data by decorrelating GO graph structure. *Bioinformatics* **22**, 1600-1607 (2006).

23. Tang H*, et al.* GOATOOLS: Tools for Gene Ontology. *Zenodo*, (2015).

24. Quinlan AR. BEDTools: The Swiss-Army Tool for Genome Feature Analysis. *Current protocols in bioinformatics / editoral board, Andreas D Baxevanis [et al]* **47**, 11 12 11-34 (2014).

25. Des Marais DL, Skillern WD, Juenger TE. Deeply diverged alleles in the Arabidopsis AREB1 transcription factor drive genome-wide differences in transcriptional response to the environment. *Mol Biol Evol* **32**, 956-969 (2015).

26. Kim D, Langmead B, Salzberg SL. HISAT: a fast spliced aligner with low memory requirements. *Nat Methods* **12**, 357-360 (2015).

27. Liao Y, Smyth GK, Shi W. featureCounts: an efficient general purpose program for assigning sequence reads to genomic features. *Bioinformatics* **30**, 923-930 (2014).

28. Love MI, Huber W, Anders S. Moderated estimation of fold change and dispersion for RNA-seq data with DESeq2. *Genome biology* **15**, 550 (2014).

29. Davidson RM*, et al.* Comparative transcriptomics of three Poaceae species reveals patterns of gene expression evolution. *Plant J* **71**, 492-502 (2012).

30. Contreras-Moreira B*, et al.* Analysis of plant pan-genomes and transcriptomes with GET_HOMOLOGUES-EST, a clustering solution for sequences of the same species. *Frontiers in Plant Science* **8**, (2017).

31. Sievers F*, et al.* Fast, scalable generation of high-quality protein multiple sequence alignments using Clustal Omega. *Molecular systems biology* **7**, 539 (2011).

32. Contreras-Moreira B, Sachman-Ruiz B, Figueroa-Palacios I, Vinuesa P. primers4clades: a web server that uses phylogenetic trees to design lineage-specific PCR primers for metagenomic and diversity studies. *Nucleic Acids Res* **37**, W95-W100 (2009).

33. Yang Z. PAML 4: phylogenetic analysis by maximum likelihood. *Mol Biol Evol* **24**, 1586-1591 (2007).

34. Wu TD, Watanabe CK. GMAP: a genomic mapping and alignment program for mRNA and EST sequences. *Bioinformatics* **21**, 1859-1875 (2005).

35. Stamatakis A. RAxML version 8: a tool for phylogenetic analysis and post-analysis of large phylogenies. *Bioinformatics* **30**, 1312-1313 (2014).

36. Darriba D, Taboada GL, Doallo R, Posada D. jModelTest 2: more models, new heuristics and parallel computing. *Nat Methods* **9**, 772 (2012).

37. Guindon S, Gascuel O. A simple, fast, and accurate algorithm to estimate large phylogenies by maximum likelihood. *Syst Biol* **52**, 696-704 (2003).

38. Pritchard JK, Stephens M, Donnelly P. Inference of population structure using multilocus genotype data. *Genetics* **155**, 945-959 (2000).

39. Earl DA, vonHoldt B. STRUCTURE HARVESTER: a website and program for visualizing STRUCTURE output and implementing the Evanno method. . *Conservation Genetics Resources* 359-361 (2012).

40. Rosenberg NA. DISTRUCT: a program for the graphical display of population structure. *Molecular Ecology Notes* **4**, 137-138 (2004).

41. Danecek P*, et al.* The variant call format and VCFtools. *Bioinformatics* **27**, 2156-2158 (2011).

42. Kearse M*, et al.* Geneious Basic: An integrated and extendable desktop software platform for the organization and analysis of sequence data. *Bioinformatics* **28**, 1647-1649 (2012).

43. Ream TS*, et al.* Interaction of photoperiod and vernalization determines flowering time of Brachypodium distachyon. *Plant physiology* **164**, 694-709 (2014).

44. Paradis E, Claude J, Strimmer K. APE: Analyses of Phylogenetics and Evolution in R language. *Bioinformatics* **20**, 289-290 (2004).

45. Quinlan AR, Hall IM. BEDTools: a flexible suite of utilities for comparing genomic features. *Bioinformatics* **26**, 841-842 (2010).

46. Huo N*, et al.* Comparison of a high-density genetic linkage map to genome features in the model grass Brachypodium distachyon. *TAG Theoretical and applied genetics Theoretische und angewandte Genetik* **123**, 455-464 (2011).
